# Does Climate Change Pose a Threat to the Guild Mimicry System of Australian Orchids?

**DOI:** 10.1002/ece3.70633

**Published:** 2024-12-09

**Authors:** Marta Kolanowska, Daniela Scaccabarozzi

**Affiliations:** ^1^ Department of Geobotany and Plant Ecology, Faculty of Biology and Environmental Protection University of Lodz Lodz Poland; ^2^ Department of Ecology and Genetics Uppsala University Uppsala Sweden; ^3^ School of Molecular and Life Sciences Curtin University Bentley Western Australia Australia

**Keywords:** bees, climatic refugia, floral mimicry, global warming, magnet species, Orchidaceae, pollination, species conservation

## Abstract

Global warming is one of the biggest threats to global biodiversity causing not only changes in the patterns of precipitation and temperature but also disturbing ecological interactions. The aim of our study was to forecast the effect of climate change on the distribution of food‐deceptive orchid species whose pollination strategy relies on a strict association with pollinators and co‐occurring rewarding Faboideae plants. We used the ecological niche modeling approach to evaluate future overlap of the suitable niches of studied orchid species with the predicted distribution of their ecological partners. Models were made based on two different global circulation models (FIO, CNRM). CNRM projections predict expansion of orchids' geographical range. In contrast, FIO prediction is less optimistic, forecasting species range contraction. The studied Faboideae species showed different responses to predicted global warming with no consistent patterns in how their suitable niches might change. Most climate change projections and scenarios of the future modifications of temperature and precipitation patterns do not predict significant loss of suitable niches of *Trichocolletes* bees (Colletidae) pollinating *Diuris* species. However, global warming has the potential to disrupt interactions between the studied orchids and their co‐occurring pea plants by altering the overlap of their geographical ranges which can further disturb pollination success. CNRM projections predict an overall loss of Faboideae within the potential geographical range of *Diuris brumalis*. Conversely, FIO projections suggest a less extensive predicted divergence. Our simulations offer suggestions for conservation strategies of orchids and potentially for other species that have a similar pollination strategy. The areas indicated here as suitable in the future for the occurrence of all ecological partners can be important climate refugia to consider in local conservation plans. The approach used in our study can serve as a model for understanding the potential effects of climate change on the strength of the pollination system via food deception.

## Graphical abstract

In this study we forecast the effect of climate change on the distribution of food‐deceptive orchid species whose pollination strategy relies on a strict association with pollinators and co‐occurring rewarding model plants. The approach used in our study can serve as a model for understanding the potential effects of climate change on the strength of plants ecological network.

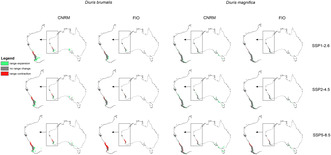

## 1 Introduction

Global warming is one of the most ubiquitous threats to global biodiversity (Urban, 2015) causing numerous abiotic (salinity, drought and UV‐B radiation (Puja and Arup Kumar 2023)) and biotic alterations (e.g., competition and facilitation, herbivory and pollination, mycorrhiza, parasitism, mutualism) (Blois et al. 2013) which can further accelerate biodiversity crisis. These include climatic niche shifts (Guisan et al. 2014), increase of biological invasions (Mainka and Howard 2010; Merow, Wilson, and Jetz 2017), alteration of ecosystem function (Grimm et al. 2013) and disruption of ecological interactions (Fontúrbel et al. 2021) which are crucial for long‐term survival of any species. Convincing proof indicates that plant species change their distribution, flowering phenology, even some risk the extinction or have already become extinct in response to climate shifts (Lavergne, Molina, and Debussche 2006; Root et al. 2005; Visser and Holleman 2001; Walther, Beißner, and Pott 2005; Zavaleta et al. 2003).

In flowering plants requiring animal vectors for sexual reproduction, the response to climate shifts can vary according to plant and the animal counterpart adaptability. – Changes in the distribution of suitable niches can cause the expansion of geographical ranges for both, or reduce the availability of pollinators for certain plants when range shifts in pollinators and plants result in a spatiotemporal mismatch. Over the last century, plants and their pollinators have appeared to respond similarly to the increase of the mean global temperature in temperate zones, both advancing the flowering phenology and the foraging season respectively (Memmott et al. 2007). On the other hand, change in the seasonal timing of flower production (i.e., phenology) can lead to mismatch with pollinator foraging season, altering the opportunity for interaction between the plants and animals (Harrison 2000; Wall, Timmerman‐Erskine, and Boyd 2003) and thus the chance of plant reproduction. Noteworthy, the fragile relationships between the species can be seriously altered before scientists can notice significant changes in the abundance and/or occurrence of individual species (Fontúrbel et al. 2021).

Global circulation models (GCMs) have been used to evaluate the impact of climate change on the distribution of various organisms, including endangered, rare (Bennett et al. 2019) and invasive species (Cho et al. 2022), and to localize climatic refugia which should be included in local conservations planning (Brambilla et al. 2022). However, little focus has been given so far to predict the complex plant pollinator relationships and other types of ecological associations in the context of global warming (Kolanowska and Michalska 2023; Kolanowska, Michalska, and Konowalik, 2021; Kolanowska, Rewicz, and Nowak, 2021). At a general ecological level, little is known about the impact of climate change on interacting species and ecosystem services such as pollination (Moss and Evans 2022). Global climate models are important tools used to predict the response of the earth's climate to different trajectories of anthropogenic greenhouse gas emissions (Wootten et al. 2017). The GCMs are based on documented physical processes to simulate the transfer of energy and materials through the global climate system. In 2021, the Intergovernmental Panel on Climate Change (IPCC) released its Sixth Assessment Report on climate change, introducing the concept of shared socioeconomic pathways (SSPs) as scenarios for projecting global socioeconomic changes until the year 2100. These trajectories describe the world with temperatures raised for 3°C–5°C above pre‐industrial levels by 2100 (Hausfather and Peters 2020) and they are linked to climate policies to generate different outcomes with radiative forcing of 1.9–8.5 watts per square meter in 2100 (Hausfather and Peters 2020).

Accounting ~28,000 species, Orchidaceae are among the most endangered plant groups in the world (Fay 2018). The diverse, broadly distributed representatives of the orchid family are ideal biological systems for modeling complex ecological relationships as the distribution of these plants are influenced by a variety of ecological factors—for example, pollinator, mycorrhizal associations, and phorophyte (host) specificity (Li et al. 2021). Interestingly, orchids have been proposed to act as ecological indicators of ecosystem health in disturbed habitats subject to anthropogenic alteration (Allen et al. 2019; Newman 2009). Indicative parameters include orchid growth, abundance, and presence. For instance, the presence and abundance of the White Fringed Orchid ( *Platanthera blephariglottis* ) in Canada were positively linked to the level of integrity of peatlands and negatively correlated with peatland size. This suggests that this species is most abundant and frequent at sites that are less influenced by human activities and the use of White Fringed Orchid as an ecological indicator may help identify peatlands worthy of conservation (Laroche, Pellerin, and Brouillet 2012). Approximately one‐third of orchids do not offer any reward to pollinators and deceive them through various pollination strategies (Ackerman et al. 2023). Generally, deceptive orchids have one or few specialized pollinators capable of effectively removing and depositing the orchid pollinia (Ackerman et al. 2023; Baguette et al. 2020; Cozzolino and Scopece 2008). Most of deceptive orchids lure their pollinators by specifically resembling other rewarding plants, model plants (food mimicry or Batesian mimicry) or general traits present in the flora community (generalized food deception) (Jersáková, Johnson, and Jürgens 2009; Johnson and Schiestl 2016).

Beyond relying on their specific pollinator, food‐deceptive orchids are influenced by the presence of other rewarding model plants. Food mimicry involves the interaction of three main actors: the mimic (orchid), the model (rewarding plants) and the receiver (pollinator) (Johnson and Schiestl 2016; Dalziell et al. 2016). For their complex reproductive biology, food‐deceptive orchids have relatively lower pollination rates comparing to rewarding or sexual deceptive orchids (Scaccabarozzi, Galimberti, Dixon, and Cozzolino, 2020; Scopece et al. 2010) and for their interaction with other organisms, orchids have the potential to indicate the occurrence of model plants and pollinating bees, providing information on the strength of the ecological network. Considering that global warming is a significant contributor to the decline of pollinators (Memmott et al. 2007), the effect of climate change on these species can be detrimental, warranting proactive measures for prevention of the decline of the single species but also of its ecological network. While numerous studies have explored the effects of global warming on orchids, only a limited number have incorporated analyses of the ecological partners of orchids, such as pollinators, phorophytes, mycorrhizal fungi, or model species (Kolanowska 2021, 2023; Kolanowska, Michalska, and Konowalik, 2021). Most existing assessments suggest that the ecological relationships within Orchidaceae may be jeopardized by climate change, potentially disrupting the future coexistence of symbiotic organisms (Evans, Janssens, and Jacquemyn 2020).

*Diuris* Sm. is a genus of food‐deceptive Australian orchids, commonly known as donkey orchids. Most donkey orchids have flowers that bear a resemblance to Australian native “egg and bacon” pea flowers (classified in tribes Bossiaeae and Mirbeliae), with which they are frequently sympatric (Indsto 2009). The species *Diuris brumalis* and *D* *iuris magnifica* in the genus mimic a range of co‐flowering pea plants to lure native bee pollinators of the genus *Trichocolletes* (Scaccabarozzi et al. 2018, Scaccabarozzi, Dixon, et al. 2020, Scaccabarozzi, Guzzetti, et al. 2020). Because of their strict relationship with pollinators displaying a typical behavior associated with the pollination of pea plants and because their reproductive success relies on the abundance and presence of pea plants, these species offer an ideal study system to test the potential effects of global warming on the distribution of the orchids in interaction with other ecological factors (i.e., pollinators and model plants).

Our study aim is to predict the effect of climate change on the distribution of the orchid species which are pollinated by mimicry of co‐occurring species. To do this, we will estimate the future overlap of the distribution of *D. brumalis* and *D. magnifica* with the predicted distribution of their pollinators and of their model pea plants.

## 2 Material and Methods

### 2.1 Studied Biological System

In our study two *Diuris* species were investigated—*D. brumalis* D.L. Jones and *D. magnifica* D.L. Jones. *Diuris brumalis*, commonly known as the winter donkey orchid, is a tuberous geophyte winter flowering plant, which is common in Darling Range near Perth, Western Australia (Scaccabarozzi et al. 2018, Scaccabarozzi, Galimberti, Dixon, and Cozzolino, 2020). Flowers of this species are self‐compatible and the pollen vector is required for pollination (Scaccabarozzi et al. 2018). Among numerous insects visiting *D. brumalis* flowers, only species of *Trichocolletes* and occasionally western honeybee ( *Apis mellifera* ) were so far observed with orchid pollinia attached to the frontal region of the insect head (Scaccabarozzi et al. 2018). However, deposition of pollinia on subsequently visited flowers was observed only in the case of *Trichocolletes* (Scaccabarozzi et al. 2018). *Diuris brumalis* shares two pollinators, *Trichocolletes capillosus* and *T. leucogenys* (Hymenoptera: Colletidae), with co‐flowering *Daviesia* Sm. (Faboideae) (Scaccabarozzi et al. 2018, Scaccabarozzi, Galimberti, Dixon, and Cozzolino, 2020). As proved by Scaccabarozzi et al. *D. brumalis* is pollinated by mimicry of co‐occurring Faboideae species and it was suggested that this relationship represents a guild mimicry case (Scaccabarozzi et al. 2018). The second species, *Diuris magnifica* (commonly called the large pansy orchid), is most probably closely allied to *D. brumalis* (Brown et al. 2013). It is distributed along the southern Western Australian coast and grows in *Banksia* and Sheoak woodland (Brown et al. 2013) which are characterized by an abundance of co‐flowering Faboideae species. This orchid flowers from August to September (Brown et al. 2013). It was confirmed to be pollinated by *Trichocolletes gelasinus* and *T. platyprosopis* (Scaccabarozzi, Guzzetti, et al. 2020). Pollination of *D. magnifica* is related with the presence of *Daviesia divaricata* (Scaccabarozzi, Guzzetti, et al. 2020).

### 2.2 List of Localities

A database of the orchid, co‐occurring Faboideae and pollinators localities was compiled based on public databases (e.g., the Global Biodiversity Information Facility, Atlas of Living Australia) and literature data (Batley and Houston 2012). The datasets with the initial number of localities for each species are provided in Annex S1. The initial dataset was verified and duplicate records for localities were removed and only records georeferenced with the precision of at least 1 km as estimated in GBIF were retained (Annex S2). Spatial thinning was done using SDMtoolbox 2.3 for ArcGIS (Brown 2014; Brown, Bennett, and French 2017) in order to reduce the spatial bias of the samples (Kramer‐Schadt et al. 2013; Tourne et al. 2019; Veloz 2009). For this process the topographic heterogeneity of the study area was calculated (Luoto and Heikkinen 2008) and reclassified into five classes of equal interval with the break values of 0.2 (low), 0.4 (medium low), 0.6 (medium), 0.8 (medium high) and 1 (high heterogeneity). Records of studied species were spatially filtered at 5.0, 10.0, 15.0, 20.0 and 25.0 km in areas of high, medium high, medium, medium low and low heterogeneity, respectively. Analyses were limited to 10.5°–43.6°S and 112.9°–153.6° E. The records used in the analyses are listed in Annex S3 in Supporting Information.

### 2.3 Ecological Niche Modeling

MaxEnt version 3.3.2 (Babar et al. 2012; Elith et al. 2011) was used for ecological niche modeling (ENM) based on presence‐only observations. The analyses were based on bioclimatic variables in 30 arc‐seconds of interpolated climate surface downloaded from WorldClim v. 2.1 (Fick and Hijmans 2017). The correlations between the bioclimatic layers was evaluated using Pearsons' correlation coefficient computed in SDMtoolbox 2.3 for ArcGIS (Brown 2014) and the variables with correlations above 0.8 were not included in the analyses (Annex S4). For removing highly correlated variables the concept of retaining layers that best represent the original input data on climate (not derived from several layers or a subset of the data) was followed. The final set of bioclimatic variables included seven layers: bio1, bio2, bio3, bio9, bio12, bio14, bio17, and bio19. Codes for these layers are provided in Annex S5. The same set of bioclimatic variables was used for all analyzed taxa.

In this study forecasts of the future distribution of the climatic niches of the species studied in 2080–2100 were made based on the GCMs developed by the First Institute of Oceanography Earth System Model (FIO‐ESM) (Bao, Song, and Qiao 2020) and by the Centre National de Recherches Météorologiques (CNRM) with Cerfacs (CNRM‐CM6) (Voldoire et al. 2019) as these GCMs were indicated as best performing models as evaluated in GCMEval (Parding et al. 2020) based on a trade off between good performance in the past and projected climate change in the focus region.

Three SSPs: SSP1‐2.6, SSP2‐4.5, and SSP5‐8.5 (Guivarch, Rozenberg, and Schweizer 2016; van Vuuren et al. 2017) were analyzed. The SSP1‐2.6 is a 2°C is a “sustainability” socioeconomic, scenario whose nameplate 2100 radiative forcing level is 2.6 W/m^−2^. The SSP2‐4.5 of the “middle of the road” socioeconomic scenario corresponds to 4.5 W/m − 2 radiative forcing level by 2100. The SSP5‐8.5 represents the upper edge of the SSP scenario spectruand m for a high fossil‐fuel using area of the world throughout the 21st century with 8.5 W/m −2 radiative forcing level by 2100 (Meinshausen et al. 2020; Schandl et al. 2020; van Vuuren et al. 2017).

In all analyses the maximum number of iterations was set to 10,000 and convergence threshold to 0.00001. A neutral (= 1) regularization multiplier value and auto features were used. All samples were added to the background even if they had combinations of environmental values that are already present in the background. The “random seed” option, which provided a random test partition and background subset for each run was applied. Twenty percent of the samples were used as test points. The run was performed as a bootstrap with 100 replicates and the output was set to logistic. Other Maxent's parameters were not modified and the default settings were used. All operations on GIS data were done using ArcGis 10.8.2 (Esri, Redlands, CA, USA). In addition, in order to avoid extrapolations outside the training data's environmental range, the “fade by clamping*”* function, which removes heavily clustered pixels from the final predictions in MaxEnt, was enabled (Owens et al. 2013; Phillips, Anderson, and Schapire 2006; Tobena et al. 2016). The evaluation of the models was done using the area under the curve (AUC) (Mason and Graham 2002) and True Skill Statistic (TSS) (Čengić et al. 2020; Shabani, Kumar, and Ahmadi 2016). The jackknife test was used to detect the most important bioclimatic variables that shape the species' geographic range (Convertino et al. 2012).

To evaluate the bioclimatic tolerances of the orchid and co‐occurring Faboideae studied the predicted niche occupancy profiles (PNOs) were created using the Phyloclim package (Heibl and Calenge 2013). These profiles combine probability of species occurrence derived from ENM and the response of each species to a given environmental variable.

SDMtoolbox v. 2.3 for ArcGIS (Brown 2014; Brown, Bennett, and French 2017) was used to calculate and visualize predicted changes in the distribution of suitable niches of species studied caused by global warming by comparing models created for “present” time and future climate projections. For this operation created models (for present‐time and future) were converted into binary rasters in Goode homolosine projection. The max Kappa value was used as a presence threshold and used to compare the extent and location of suitable niches of studied species between present‐time and future models. To calculate the overlap between studied ecological partners we determined the coverage of the areas which will be suitable for the occurrence of analyzed species using their modeled potential geographical ranges.

## 3 Results

### 3.1 Models' Evaluation and Limiting Factors

The model performance indexes (AUC and TSS) are presented in Annex S6 together with max Kappa value which was used as a presence threshold. All created models received good scores on both AUC (0.975–0.997) and TSS (0.817–0.985) tests. Also, the specificity of constructed simulations was high, ranging from 0.892 to 0.985.

The results of the jackknife test (Annex S7) of variable importance showed that for *D. brumalis* and *D. magnifica* , bio19 (precipitation of the coldest quarter) was the environmental variable with highest gain when used in isolation and having the most useful information by itself. The mean temperature of the driest quarter (bio9) exhibited the most significant reduction in gain when omitted as an environmental variable, suggesting that it contained unique information not captured by other variables in the models of *D. brumalis*. In case of *D. magnifica* bio2 (mean diurnal range) was more important in this aspect.

Among all *Daviesia* species, bio19 contained the most valuable information on its own. In models of *T. gelasinus* and *T. leucogenys* bio19 has the highest gain when used in isolation and the same variable decreases the gain the most when it is omitted. As calculated, for *T. platyprosopis* bio9 has the most useful information by itself and bio19 has the most information that isn't present in the other variables.

Created PNO profiles (Annex S8) showed that all studied species are generally characterized by similar ecological tolerances in respect to bioclimatic variables studied and no significant differences in their preferences were identified.

### 3.2 Impact of Climate Change on Orchid, Co‐Occurring Faboideae and Pollinators

**FIGURE 1:**
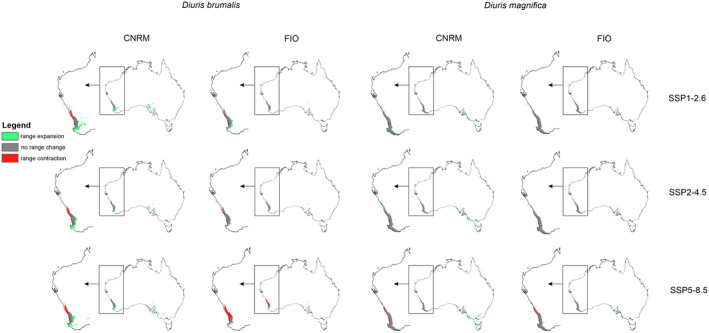
Changes in orchids (*Diuris brumalis* and *D. magnifica* ) distribution according to the CNRM and FIO projections in three various climate change scenarios.

**FIGURE 2:**
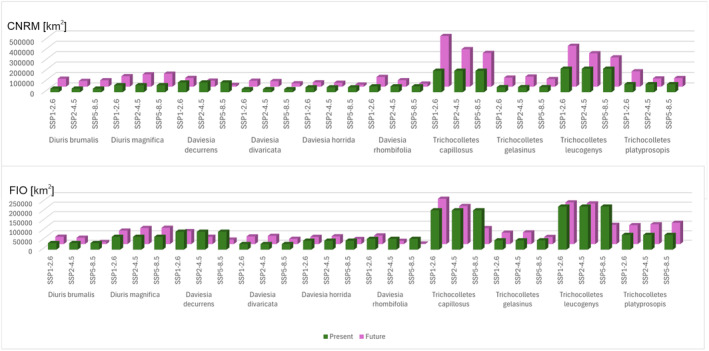
Barplots visualizing changes in the coverage of suitable niches of studied species, *Diuris* and Faboideae species.

CNRM projections indicated that climate change will be generally favorable for studied orchids (Figures 1 and 2, Table 1). *Diuris brumalis* will expand its range by 122.91% in the less harmful CNRM SSP1‐2.6 scenario. In the same scenario projected by FIO the expansion will be lower reaching just 12.59%. In the least beneficial for *D. brumalis* scenario projected by CNRM (SSP2‐4.5), the orchid range will also expand (for 61.64%). FIO forecast is less optimistic predicting 2% range contraction in SSP2‐4.5 scenario and as much as 67% in SSP5‐8.5 scenario. For *D. magnifica* global warming will be beneficial and the potential range of this species will expand by at least 5.42% (FIO SSP1‐2.6). In the most optimistic forecast CNRM 5–8.5 the potential expansion will reach almost 86%.

**TABLE 1:** Changes in the coverage [km^2^] of suitable niches of *Diuris* species, co‐occurring Faboideae and pollen vectors.

Ecological group	Species	Projection	Scenario	Range expansion	Present in both models	Range contraction	Change
Orchid	*Diuris brumalis*	CNRM	SSP1‐2.6	50360.69	26123.62	8204.054	+122.81%
			SSP2‐4.5	31036.48	24449.82	9877.845	+61.64%
			SSP5‐8.5	40481.14	21096.26	13231.41	+79.38%
		FIO	SSP1‐2.6	6623.394	32025.03	2302.638	+12.59%
			SSP2‐4.5	2558.106	30854.49	3473.181	−2.67%
			SSP5‐8.5	1086.811	10070.94	24256.73	−67.50%
Orchid	*Diuris magnifica*	CNRM	SSP1‐2.6	34196.94	66738.04	459.6731	+50.21%
			SSP2‐4.5	51870.44	66943.1	254.6145	+76.81%
			SSP5‐8.5	62587.31	62338.68	4859.035	+85.91%
		FIO	SSP1‐2.6	5718.573	65123.2	2074.51	+5.42%
			SSP2‐4.5	17835.83	66656.02	541.6966	+25.74%
			SSP5‐8.5	23289.53	62336.12	4861.598	+27.42%
Faboideae	*Daviesia decurrens*	CNRM	SSP1‐2.6	6119.291	77937.66	15848.47	−10.37%
			SSP2‐4.5	2851.169	55105.24	38680.89	−38.20%
			SSP5‐8.5	453.6922	15908.28	77877.85	−82.55%
		FIO	SSP1‐2.6	529.7348	67393.37	26392.76	−27.58%
			SSP2‐4.5	1019.312	37462.5	56323.63	−58.97%
			SSP5‐8.5	576.7274	22766.63	71019.49	−75.11%
Faboideae	*Daviesia divaricata*	CNRM	SSP1‐2.6	27735.89	29352.43	19.65145	+94.36%
			SSP2‐4.5	24561.75	29357.56	14.52499	+83.57%
			SSP5‐8.5	6597.762	25790.4	3581.691	+10.27%
		FIO	SSP1‐2.6	11014.21	29298.61	73.47934	+37.25%
			SSP2‐4.5	13786.78	29330.22	41.86614	+46.80%
			SSP5‐8.5	3452.675	24145.65	5226.432	−6.04%
Faboideae	*Daviesia horrida*	CNRM	SSP1‐2.6	2473.52	39348.19	7588.024	−10.90%
			SSP2‐4.5	3094.677	34689.09	12247.13	−19.50%
			SSP5‐8.5	586.9803	19941.1	26995.11	−56.26%
		FIO	SSP1‐2.6	31.61321	37871.77	9064.446	−19.24%
			SSP2‐4.5	120.4719	40863.91	6072.299	−12.68%
			SSP5‐8.5	28.19556	26675.57	20260.65	−43.11%
Faboideae	*Daviesia rhombifolia*	CNRM	SSP1‐2.6	47295.07	46410.75	10007.72	+66.09%
			SSP2‐4.5	27590.64	34657.47	21760.99	+10.33%
			SSP5‐8.5	11150.06	18697.08	37721.39	−47.10%
		FIO	SSP1‐2.6	1305.54	44092.73	12325.73	−19.53%
			SSP2‐4.5	210.1851	16892.56	39525.91	−69.69%
			SSP5‐8.5	5.126466	2775.981	53642.48	−95.07%
Pollen vecor	*Trichocolletes capillosus*	CNRM	SSP1‐2.6	292136.8	195114.1	11236.36	+136.13%
			SSP2‐4.5	204227.3	156493.9	49856.59	+74.81%
			SSP5‐8.5	206050.6	118474.3	87876.17	+57.27%
		FIO	SSP1‐2.6	53655.3	184393.9	21956.65	+15.36%
			SSP2‐4.5	32693.18	167196.3	39154.24	−3.13%
			SSP5‐8.5	2587.156	81217.74	125132.8	−59.39%
Pollen vecor	*Trichocolletes gelasinus*	CNRM	SSP1‐2.6	38902.19	48287.04	388.757	+79.12%
			SSP2‐4.5	49419.99	47818.82	856.9742	+99.77%
			SSP5‐8.5	31851.59	41254.38	7421.414	+50.19%
		FIO	SSP1‐2.6	11732.77	48219.54	456.2555	+23.17%
			SSP2‐4.5	13467.23	48019.61	656.1876	+26.32%
			SSP5‐8.5	4029.402	33478.39	15197.41	−22.94%
Pollen vecor	*Trichocolletes leucogenys*	CNRM	SSP1‐2.6	167990.9	224886.9	964.63	+73.95%
			SSP2‐4.5	118499.1	202981.6	22870.02	+42.34%
			SSP5‐8.5	133466.7	148584.6	77266.95	+24.88%
		FIO	SSP1‐2.6	23952.56	194856.1	30995.47	−3.12%
			SSP2‐4.5	31012.56	182420.2	43431.42	−5.50%
			SSP5‐8.5	4706.95	96198.99	129652.6	−55.32%
Pollen vecor	*Trichocolletes platyprosopis*	CNRM	SSP1‐2.6	71639.8	76194.66	928.7447	+91.69%
			SSP2‐4.5	10525.49	68825.37	8298.039	+2.89%
			SSP5‐8.5	20992.02	61785.02	15338.39	+7.33%
		FIO	SSP1‐2.6	22911.88	76546.68	576.7274	+28.96%
			SSP2‐4.5	28607.39	75431.67	1691.734	+34.90%
			SSP5‐8.5	38489.51	73262.32	3861.083	+44.90%

Faboideae species will respond variously to predicted global warming (Table 1, Annex S9). *Daviesia decurrens* will face habitat loss. In the most optimistic scenario SSP1‐2.6 (CNRM) the potential range of this species will be 10% smaller than currently observed. In the worst‐case scenario (SSP5‐8.5, CNRM) *D. decurrens* will be threatened by extinction by losing 83% of suitable niches. *Daviesia divaricata* will most probably benefit from climate changes and in all CNRM and most of FIO projections the potential range of this species will expand. The only scenario which is less optimistic for this plant is FIO SSP5‐8.5 in which the species will lose about 6% of currently suitable niches. All analyzed scenarios indicated habitat loss also for *Daviesia horrida* which will lose between 11% (SSP1‐2.6, CNRM) and 56% (SSP5‐8.5, CNRM) of suitable niches. CNRM projection showed various scenarios for *Daviesia rhombifolia* which will expand its potential range in SSP1‐2.6 and SSP2‐4.5 scenarios (66% and 10% respectively) but will face significant (47%) habitat loss in SSP5‐8.5 scenario. FIO projection indicated only potential range contraction of *D. rhombifolia* in all analyzed scenarios (20%–95%).

Generally, most projections and scenarios are optimistic for *Trichocolletes* species (Table 1, Annex S10). All CNRM projections of three analyzed scenarios predict potential expansion of studied bees. FIO forecasts are inconclusive. In FIO SSP1‐2.6 *T. capillosus* will slightly increase its geographical range, while in two other FIO scenarios this species will lose 3%–59% of currently available niches. According to calculated range changes, new areas will become suitable for *T. gelasinus* occurrence in FIO SSP1‐2.6 and SSP2‐4.5 scenarios. In FIO SSP5‐8.5 scenario this bee will face habitat loss (22%). All three FIO scenarios will be damaging for *T. leucogenys* which will lose 3%–55% of suitable niches. *Trichocolletes platyprosopis* will benefit from climate changes in all analyzed FIO scenarios (28%–44%).

### 3.3 Future Overlap of Suitable Niches of Orchids and Co‐Occurring Faboideae

**FIGURE 3:**
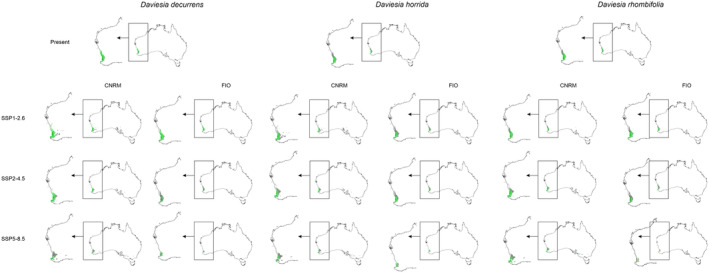
Overlap of potential range of *Daviesia* species and *Diuris brumalis*. Areas of overlap marked with green, areas suitable only for orchid occurrence marked in gray.

**FIGURE 4:**
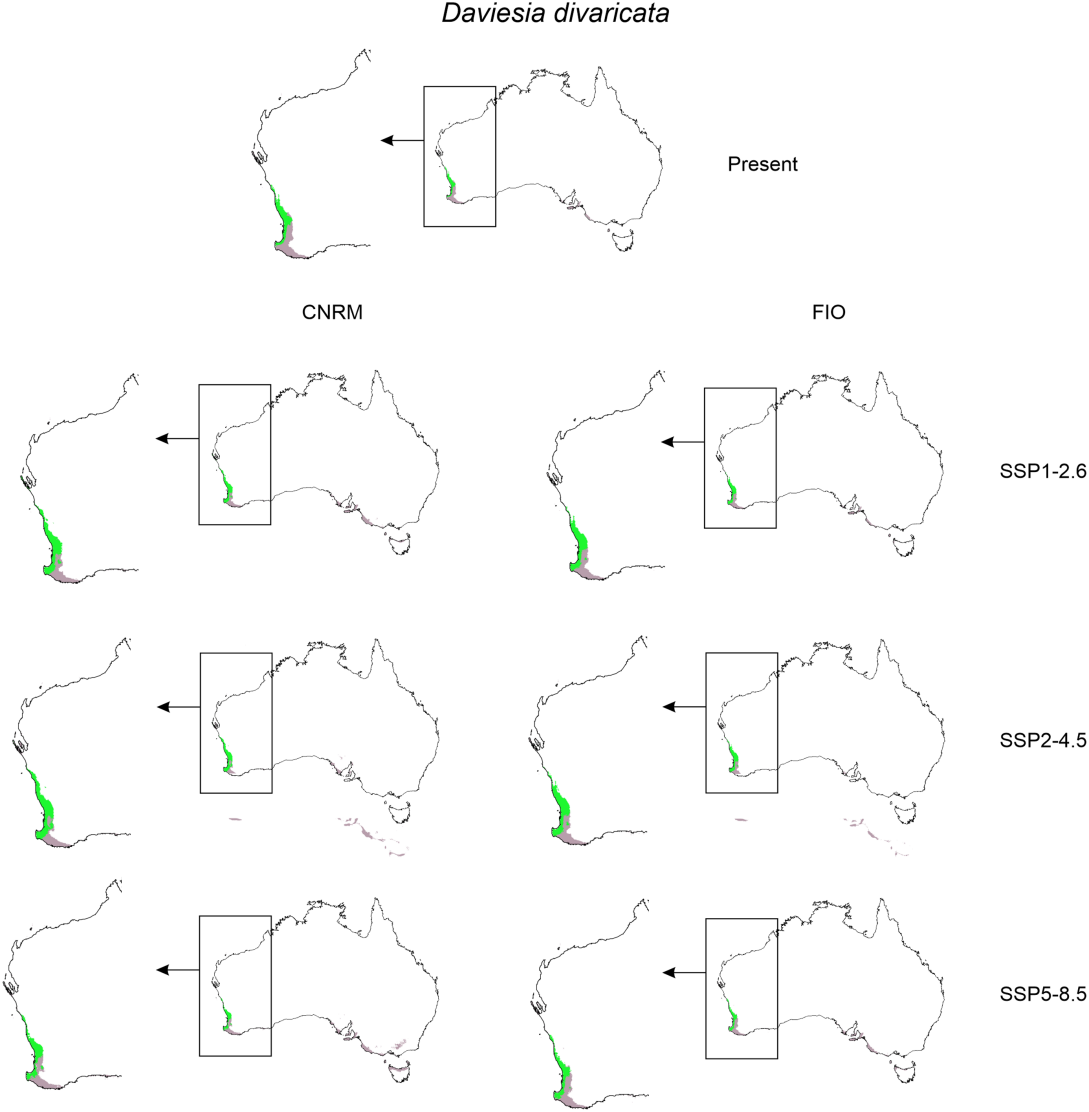
Overlap of potential range of *Daviesia divaricata* and *Diuris magnifica*. Areas of overlap marked with green, areas suitable only for orchid occurrence marked in gray.

Global warming can affect interactions of studied orchids with their co‐occurring Faboideae species (Figures 3 and 4, Table 2) as the climate change will change the overlap between *Diuris* and pea plants geographical ranges. In CNRM projection the overall loss of Faboideae within *D. brumalis* potential geographical range is predicted. While currently *D. decurrens* is available in 98% of *D. brumalis* potential range, the future overlap between these species will reach 20%–53%. A similar decrease will be observed for *D. horrida* and *D. rhombifolia*. In FIO projections the predicted divergence will be of lesser extent. *D. decurrens* will be available in 36%–91% of *D. brumalis* range, *D. horrida* in 46%–64%, and *D. rhombifolia* in 24%–65%. In the case of *D. magnifica* the loss of overlap between the orchid and *Daviesia divaricata* will not change significantly.

**TABLE 2:** Changes in the overlap of suitable niches between orchids studied and co‐occurring Faboideae.

Orchid species	Faboideae species	Present	CNRM			FIO		
			SSP1‐2.6	SSP2‐4.5	SSP5‐8.5	SSP1‐2.6	SSP2‐4.5	SSP5‐8.5
**Australia–whole territory**								
*Diuris brumalis*	*Daviesia decurrens*	**98.14%**	53.01%	56.76%	20.44%	91.03%	36.00%	62.61%
	*Daviesia horrida*	**58.86%**	32.62%	40.44%	20.29%	46.43%	52.67%	64.95%
	*Daviesia rhombifolia*	**69.27%**	50.87%	46.36%	34.01%	65.51%	38.11%	24.53%
*Diuris magnifica*	*Daviesia divaricata*	**31.37%**	33.27%	31.28%	18.16%	39.49%	36.08%	23.76%
**Western Australia only**								
*Diuris brumalis*	*Daviesia decurrens*	**99.96%**	81.20%	79.51%	34.68%	91.25%	36.00%	62.61%
	*Daviesia horrida*	**59.95%**	50.15%	56.67%	34.27%	46.54%	52.67%	64.95%
	*Daviesia rhombifolia*	**70.55%**	73.07%	64.02%	48.40%	65.67%	38.11%	24.53%
*Diuris magnifica*	*Daviesia divaricata*	**41.20%**	57.30%	61.40%	44.52%	53.66%	55.29%	41.69%

*Note:* Bold values indicate the current overlap between orchids and their ecological partners.

Considering exclusively Western Australia a general decrease of overlap between *D. brumalis* and co‐occurring Faboideae is expected. Only in FIO SSP5‐8.5 and CNRM SSP1‐2.6 scenarios a small increase of *D. horrida* and *D. rhombifolia* availability is predicted respectively.

### 3.4 Future Overlap of Suitable Niches of Orchids and Their Pollinators

**FIGURE 5:**
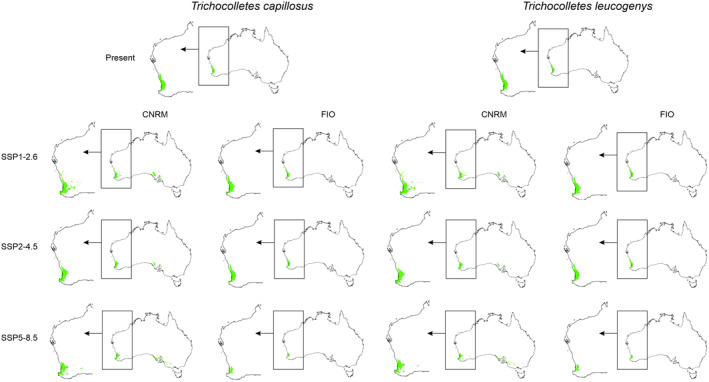
Overlap of potential range of pollinators, *Thrichocolletes* bees, and *Diuris brumalis*. Areas of overlap marked with green, areas suitable only for orchid occurrence marked in gray.

**FIGURE 6:**
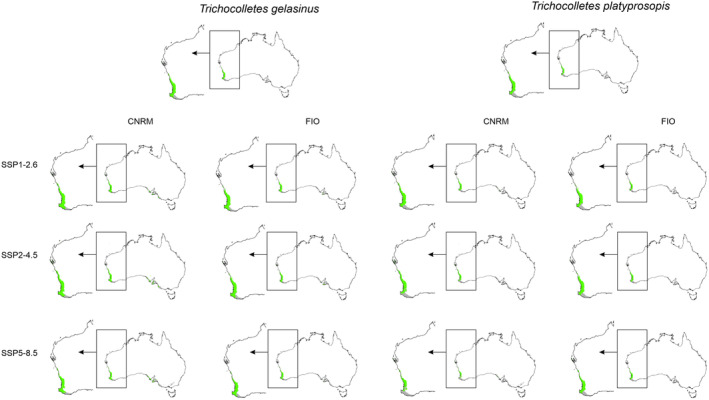
Overlap of potential range of pollinators, *Trichocolletes* bees, and *Diuris magnifica*. Areas of overlap marked with green, areas suitable only for orchid occurrence marked in gray.

Global warming can disturb interactions of studied orchids with their pollinators (Figures 5, 6, Table 3) by changing the availability of insects for *Diuris*. Considering the whole potential range of orchids and their pollinators only *T. capillosus* will be constantly available as pollen vector for *D. brumalis*. The overlap of orchids and both *T. leucogenys* and *T. platyprosopis* will be smaller than currently observed. The presence of *T. gelasinus* within *D. magnifica* range is uncertain. While in all CNRM projection insects will be present in 60%–61% of orchid range, two FIO scenarios (SSP2‐4.5, SSP5‐8.5) predict decrease of pollinator availability.

**TABLE 3:** Changes in the overlap of suitable niches between orchids studied and pollen vectors. Red values given in cases of overlap decline (compared to present‐time overlap).

Orchid species	Pollinator	Present	CNRM			FIO		
			SSP1‐2.6	SSP2‐4.5	SSP5‐8.5	SSP1‐2.6	SSP2‐4.5	SSP5‐8.5
**Australia–whole territory**								
*D. brumalis*	*T. capillosus*	**100.00%**	100.00%	100.00%	100.00%	100.00%	100.00%	100.00%
	*T. leucogenys*	**97.69%**	82.92%	82.92%	85.59%	85.59%	94.93%	94.93%
*D. magnifica*	*T. gelasinus*	**58.30%**	61.02%	61.02%	60.71%	60.71%	45.97%	45.97%
	*T. platyprosopis*	**52.94%**	43.49%	43.49%	33.28%	33.28%	22.23%	22.23%
**Western Australia only**								
*D. brumalis*	*T. capillosus*	**100.00%**	100.00%	100.00%	100.00%	100.00%	100.00%	100.00%
	*T. leucogenys*	**100.00%**	99.83%	99.83%	99.92%	99.92%	96.92%	96.92%
*D. magnifica*	*T. gelasinus*	**76.51%**	85.59%	85.59%	86.47%	86.47%	77.97%	77.97%
	*T. platyprosopis*	**69.53%**	72.46%	72.46%	65.08%	65.08%	50.80%	50.80%

*Note:* Bold values indicate the current overlap between orchids and their ecological partners.

More optimistic future of orchid–pollinator relationship is expected in case of Western Australia only. Within this area a small decrease of *T. leucogenys* overlap with *D. brumalis* range is predicted. Again, *T. capillosus* will be present in the whole orchid range. Considering pollinators of *D. magnifica*, in CNRM SSP 5–8.5 and FIO predictions a decrease (4%–19%) of *T. platyprosopis* availability for *D. magnifica* is forecasted. *T. gelasinus* will be present in 77%–86% of the orchid range.

## 4 Discussion

Our study predicts the impact of climate change on the distribution of two food‐deceptive orchid species, *D. brumalis* and *D. magnifica* , whose pollination strategy relies on a strict association with pollinators and co‐occurring rewarding pea *Daviesia* plants (model plants). These species are endemic and have a limited distribution in Southwestern Australia. Therefore, the predictive scenario for their distribution holds significant conservation implications for these orchids, as well as for other species within the genus in *Diuris corymbosa* complex, that likely have a similar pollination strategy (Brown et al. 2013). Moreover, our approach can serve as a model for understanding the potential effects of climate change on orchid species that employ food deception for pollination. Our simulations encompass the interactions of the orchid species with their ecological partners, including bee pollinators of the *Trichocolletes* genus and multiple species of pea plants (Faboideae).

### 4.1 General Forecast Scenario and Their Conservation Implications

According to our analyses, the two studied pollination systems will respond differently to predicted climate change, proving that even similar, closely related species inhabiting the same geographical region can respond differently to global warming. To delve into the interpretation of our findings, it is crucial to emphasize the ecological requirements of the species that inhabit distinct environments characterized by varying degrees of habitat alteration. *D. brumalis* predominantly grows in well‐preserved habitats, wild Eucalypt forests, while *D. magnifica* is confined to bushland remnants, typically found in Banksia woodland (Brown et al. 2013). The investigation into the pollination ecology of these two species revealed slight variations, indicating different responses in orchid fitness concerning the guild of model plants. The pollination of *D. brumalis* depends on two species of *Trichollete*s, namely *T. capillosus* and *T. leucogenys*, while the pollination of *D. magnifica* principally relies on *T. gelasinus* and *T. platyprosopis*. The mimicry exhibited by *D. brumalis* is highly specific to pea plant species within the *Daviesia* genus, and the orchid benefits from the abundance of these model plants (Scaccabarozzi et al. 2018). In contrast, *D. magnifica* mimics various genera of pea plants (*Daviesia, Bossiaae, Jacksonia*), and its fitness does not gain advantages from their co‐occurrence. Moreover, it is influenced by the presence of a non‐model species (*Hardenbergia comptoniana*). Despite this species is not a model for *Diuris* is a magnet species favoring, up to a certain abundance, the pollination of the orchid (Johnson et al. 2003; Scaccabarozzi, Guzzetti, et al. 2020). Despite both orchids maintaining mimicry toward pea plants, the pollination mechanism in the latter case appears more generalized compared to *D. brumalis*. The diverse forecast scenarios observed for the two species that share a similar pollination strategy, may be attributed to the interaction of multiple biotic factors incorporated by the prediction model (i.e., combined presence of different pollinators, and the coexistence of various model plants). This resulted in different forecast scenarios for the two species.

Our study confirmed the significance of analyzing not just the potential future distribution of plant species but also incorporating data on their ecological partner(s) into the projections. In the case of *Diuris*, planning conservation measures should prioritize locations of conservation areas where both Faboideae species and pollinators are able to co‐occur with the orchid species. Our predictions support the importance of applying ENM outcomes in conservation biology, for instance with threatened or rare species in face of climate change. Specifically, ENM technique can aid in spatial prioritization for biodiversity preservation (Ishihama et al. 2019) by offering insights into the spatial localization of species' suitable niches under different climate change scenarios. This is achieved through the analysis of bioclimatic variables and species' occurrence records (Gengping et al. 2013).

**FIGURE 7:**
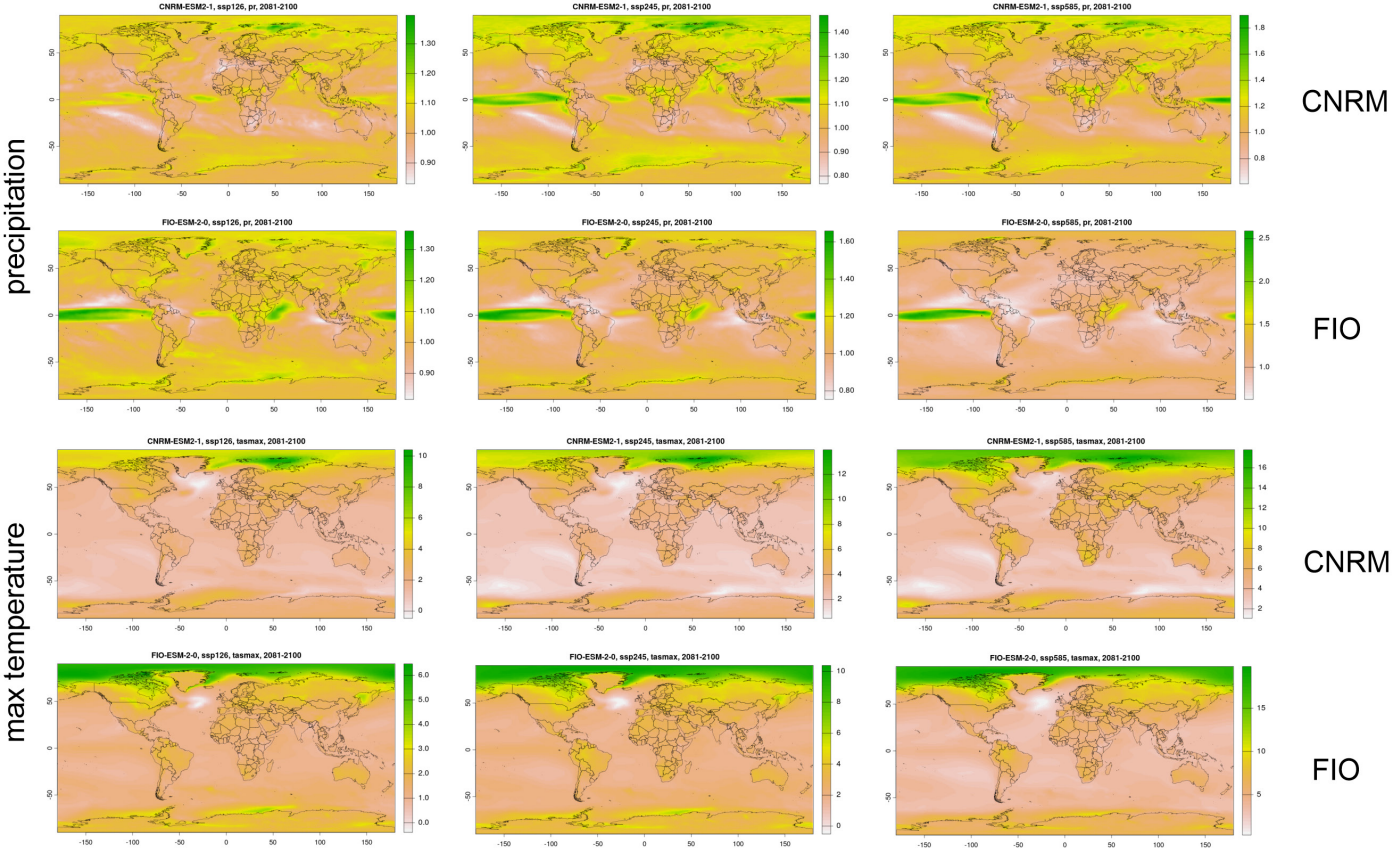
Comparison of FIO and CNRM simulations of maximum temperature and precipitation. Maps provided by (Fick and Hijmans 2017).

As any predictive simulation, our study presents limitations. We used two different GCMs which received high scores of performances in Australia. A caveat is that the models did not converge, as the predictions for changes in the distribution of suitable niches for the studied species were not identical. Generally, in comparison to CNRM, FIO forecasts predicts higher temperature in Western Australia, higher precipitation in SSP1‐2.6 and lower precipitation in SSP2‐4.5 and SSP5‐8.5 (Figure 7). The discrepancy between forecasted climatic conditions in the future between FIO and CNRM can be a result of different simulations of the Antarctic circumpolar wave (ACW), a large‐scale oceanic and atmospheric pattern in the Southern Ocean which has substantial impacts on the global climate (Lu, Zhao, and Zhou 2020). Despite dissimilarities in the extend of the range shift which is predicted to occur in studied species as a result of global warming, maps presented in this study are showing numerous areas which can serve as climate refugia for studied ecological partners in both FIO and CNRM simulations.

### 4.2 Future of *Diuris* Guild Mimicry Systems

Our analyses predict that global warming will not significantly affect *Diuris magnifica* distribution range and its guild mimicry system. *Daviesia divaricata* which is linked to the pollination success of *D. magnifica* will be available in most part (41%–61%) of the orchid distribution range. While *T. gelasinus* will expand its occurrence within *D. magnifica* distribution range (76%–86%), we expect a small decline in *T. platyprosopis* occurrence (down to 50%). However, our prediction models did not consider abiotic factors such as habitat fragmentation that affected *D. magnifica* pollination success (Scaccabarozzi et al. 2023). In fact, orchids in smaller bushland reserves experienced lower pollination success compared to orchids in bigger bushland remnants (Scaccabarozzi et al. 2024). Loss and degradation of natural habitat are the primary causes of declines in global biodiversity (Fahrig 2003; Segan, Murray, and Watson 2016). Particularly, habitat fragmentation along with climate change are one of the primary causes for the global pollinator decline of plant pollinators (Grass et al. 2018; Potts et al. 2010). Considering this, we need to underline that conservation measures deriving from climate predictions should also weigh the effect of other key interaction factors (i.e., habitat change) to apply effective conservation practices of the target species.

Our analysis presented a different forecast scenario for the congeneric species *Diuris brumalis*. This latter will more likely face loss of co‐occurring pea plant species (down to 24% in case of *Daviesia rhombifolia*) which are model plants for the orchids and therefore crucial for the pollination success of the species. At the same time a small decline in pollinator availability (*T. leucogenys*) is expected to occur (about 3%). This scenario could potentially result in the disruption of the orchid–pollinator interaction which depends on the presence of rewarding pea plants. Alternatively, it may lead to the niche occupation by other pea plants that could potentially replace the current ecological role of *Daviesia* in *D. brumalis* pollination. Currently, it is challenging to anticipate whether other pea plants will assume the role currently played by *Daviesia* and there will be a shift in ecological niche as predicted by some models forecasting plant community responses to global change (Franklin et al. 2016). While presently the flowering time of *Diuris* and *Daviesia* species is well‐synchronized with the activity time of their pollinators, some studies have indicated that climate warming can disrupt mutualistic interactions between solitary bees and plants by advancing insect phenology more rapidly than plant flowering (Schenk, Krauss, and Holzschuh 2018). This potential mismatch would pose a threat to the reproductive success of the orchid species, putting them in a precarious situation.

In this research we analyzed only future stability of the relationship between *Diuris* and *Daviesia* because these peas are considered to be the most important model plants in the studied pollination system, but also other faboid species should be studied in the future to fully understand the impact of global warming on *Diuris* sexual reproduction (Scaccabarozzi, Guzzetti, et al. 2020).

### 4.3 Other Factors Affecting Orchids and Pollinators Distribution

Our models might underestimate the effect of global change on the orchid species distribution. In fact, in our analysis we did not include at least two more ecological factors influencing *Diuris* reproductive biology and occurrence, which are fire regime and mycorrhizal associations. Donkey orchids strongly depend on seasonal conditions and flowering of these plants is enhanced after fire, especially in areas where the surrounding vegetation has grown very dense (Duncan 2012). However, climate markedly amplifies the risk of fire‐prone weather conditions (van Oldenborgh et al. 2021), making it challenging to assess if the increased frequency of fires will be beneficial for *Diuris* flowering, especially in the face of extreme fires. These could indeed be harmful, causing disruption to both the orchid's ecological partner and its underground root system.

In this regard, it is well known that dry weather and modification of soil characters may be especially dangerous for orchid mycorrhizal fungi which are required for seed germination (Rasmussen et al. 2015). Unfortunately, the models of further changes in the soil properties which caused by climate change have not been modeled and they could not be included in our simualtions. So far little is known about the specificity of *Diuris* symbiotic fungi, but available research indicated that donkey orchids associate with a narrow taxonomic range of fungi within the cosmopolitan family Tulasnellaceae (*Rhizoctonia* alliance) (Smith, James, and McLean 2010). Due to the lack of identification of species composition of mycorrhizal fungi in studied orchids, and poor georeferenced data on *Rhizoctonia* distribution it is presently not possible to evaluate the future potential distribution of *Diuris* symbiotic fungi.

In addition, while our analyses mapped future distribution of climatic niches of orchid bee pollinators, there are additional factors which can affect the actual occurrence of these insects. *Trichocolletes* are solitary, ground‐nesting bees and they mostly depend on floral and nesting resources (Houston et al. 2023). While our analyses did not indicate any significant loss of food source availability for *Trichocolletes*, the changes in soil characters cannot be predicted using solely bioclimatic data. Increased frequency and intensiveness of fires resulting from global warming can significantly affect the soil properties and inhibit *Trichocolletes* from nesting in the climatically suitable areas (Harvey et al. 2023). Conversely, there is evidence indicating that wildfires can create conditions conducive to supporting native bees and the resources essential for their prospering (Burkle et al. 2019; Galbraith et al. 2019).

Concluding, the enduring impacts of land‐use change and human‐altered fire regimes, along with natural fire events, can overshadow or interact with the impacts of climate change. Despite in our models, we did not incorporate other potential ecological factors such as fire regimes and fungi partners due to a lack of consistent information, we included main elements of the pollination ecology of the species. Therefore, approaches like ours can serve as a baseline for assessing conservation measures aimed at protecting rare or endangered orchid floral species with a similar pollination strategy. The disappearance of a species can occur silently and suddenly and anticipating the decline or collapse of a species is of utmost importance.

In fact, while many species can still propagate through vegetative means or self‐pollination, and populations of the species may be represented by several plants, the connections with ecological partners linked to their sexual reproduction can instead be at risk and disrupted. This poses a significant threat to the species' survival over time.

Since orchids have been proposed to act as ecological indicators of ecosystem health in altered landscapes, our projections may help identify and prioritize areas worthy of species conservation aimed to preserve the strength of their ecological network.

## Author Contributions

**Marta Kolanowska:** conceptualization (equal), data curation (equal), formal analysis (equal), investigation (equal), methodology (equal), project administration (equal), software (equal), supervision (equal), validation (equal), visualization (equal), writing – original draft (equal), writing – review and editing (equal). **Daniela Scaccabarozzi:** conceptualization (equal), data curation (equal), investigation (equal), resources (equal), validation (equal), writing – original draft (equal), writing – review and editing (equal).

## Conflicts of Interest

The authors declare no conflicts of interest.

## Supporting information


**Annex S1** GBIF datasets of the localities of the species studied and final number of records used in ENM analyses.
**Annex S2**. Localities of species studied.
**Annex S3**. Records used in the ENM analyses.
**Annex S4**. Pearsons’ correlation coefficient computed for 19 bioclimatic variables.
**Annex S5**. Bioclimatic variables. Layers used in ENM analyses marked with ‘+’.
**Annex S6**. Modeling performance indexes.
**Annex S7**. Results of the jackknife test of variable importance.
**Annex S8**. Predicted niche occupancy (PNO) profiles for the species studied.
**Annex S9**. Changes in the distribution of suitable niches for *Daviesia* studied.
**Annex S10**. Changes in the distribution of suitable niches for pollinators studied.
**Annex S11**. Overlap of potential range of *Daviesia* species and *Diuris brumalis*.
**Annex S12**. Overlap of potential range of *Daviesia* species and *Diuris magnifica*.
**Annex S13**. Overlap of potential range of pollinators and *Diuris brumalis*.
**Annex S14**. Overlap of potential range of pollinators and *Diuris magnifica*.

## Data Availability

The data interpreted and discussed in this paper are presented in the manuscript and Supporting Information.
