# Supplementary material for: Does Climate Change Pose a Threat to the Guild Mimicry System of Australian Orchids?
Source: Ecol Evol. 2024 Dec 9;14(12):e70633. doi: 10.1002/ece3.70633 (PMC11628745; doi:10.1002/ece3.70633)
Supplement: Supplementary file 1 — Annex S1 GBIF datasets of the localities of the species studied and final number of records used in ENM analyses. Annex S2. Localities of species studied. Annex S3. Records used in the ENM analyses. Annex S4. Pearsons’ correlation coefficient computed for 19 bioclimatic variables. Annex S5. Bioclimatic variables. Layers used in ENM analyses marked with ‘+’. Annex S6. Modeling performance indexes. Annex S7. Results of the jackknife test of variable importance. Annex S8. Predicted niche occupancy (PNO) profiles for the species studied. Annex S9. Changes in the distribution of suitable niches for Daviesia studied. Annex S10. Changes in the distribution of suitable niches for pollinators studied. Annex S11. Overlap of potential range of Daviesia species and Diuris brumalis. Annex S12. Overlap of potential range of Daviesia species and Diuris magnifica. Annex S13. Overlap of potential range of pollinators and Diuris brumalis. Annex S14. Overlap of potential range of pollinators and Diuris magnifica. [file ECE3-14-e70633-s001.docx]

**Supporting Information**

**Does climate change pose a threat to the guild mimicry system of Australian orchids?**

Marta Kolanowska & Daniela Scaccabarozzi

^Marta Kolanowska1 & Daniela Scaccabarozzi2,3^

^1^ Faculty of Biology and Environmental Protection, Department of Geobotany and Plant Ecology, University of Lodz, ul. Banacha 12/16, 90-237 Lodz, Poland

^2^ Department of Ecology and Genetics, Uppsala University, Uppsala, Sweden

^3^ School of Molecular and Life Sciences, Curtin University, Bentley, Western Australia, Australia

*Corresponding author: marta.a.kolanowska@gmail.com

**Annex S1**. GBIF datasets of the localities of the species studied and final number of records used in ENM analyses.

| **Species** | **Dataset / data source** | **Total nr of records** | **Records used in ENM analysis** |
| --- | --- | --- | --- |
| *Diuris brumalis* | https://www.gbif.org/occurrence/download/0010727-230530130749713 | 186 | 17 |
| *Daviesia decurrens* | https://www.gbif.org/occurrence/download/0010734-230530130749713 | 858 | 69 |
| *Daviesia horrida* | https://www.gbif.org/occurrence/download/0010739-230530130749713 | 385 | 26 |
| *Daviesia rhombifolia* | https://www.gbif.org/occurrence/download/0010746-230530130749713 | 215 | 28 |
| *Daviesia divaricata* | https://www.gbif.org/occurrence/download/0045242-231002084531237 | 340 | 27 |
| *Diuris magnifica* | https://www.gbif.org/occurrence/download/0045232-231002084531237 | 235 | 13 |
| *Trichocolletes capillosus* | Batley & Houston, 2012. Revision of the Australian bee genus Trichocolletes Cockerell (Hymenoptera: Colletidae: Paracolletini). Records of the Australian Museum 64(1): 1–50. | 10 | 8 |
| *Trichocolletes gelasinus* | Batley & Houston, 2012. Revision of the Australian bee genus Trichocolletes Cockerell (Hymenoptera: Colletidae: Paracolletini). Records of the Australian Museum 64(1): 1–50. | 20 | 14 |
| *Trichocolletes leucogenys* | Batley & Houston, 2012. Revision of the Australian bee genus Trichocolletes Cockerell (Hymenoptera: Colletidae: Paracolletini). Records of the Australian Museum 64(1): 1–50. | 40 | 34 |
| *Trichocolletes platyprosopis* | Batley & Houston, 2012. Revision of the Australian bee genus Trichocolletes Cockerell (Hymenoptera: Colletidae: Paracolletini). Records of the Australian Museum 64(1): 1–50. | 6 | 4 |

**Annex S2**. Localities of species studied.


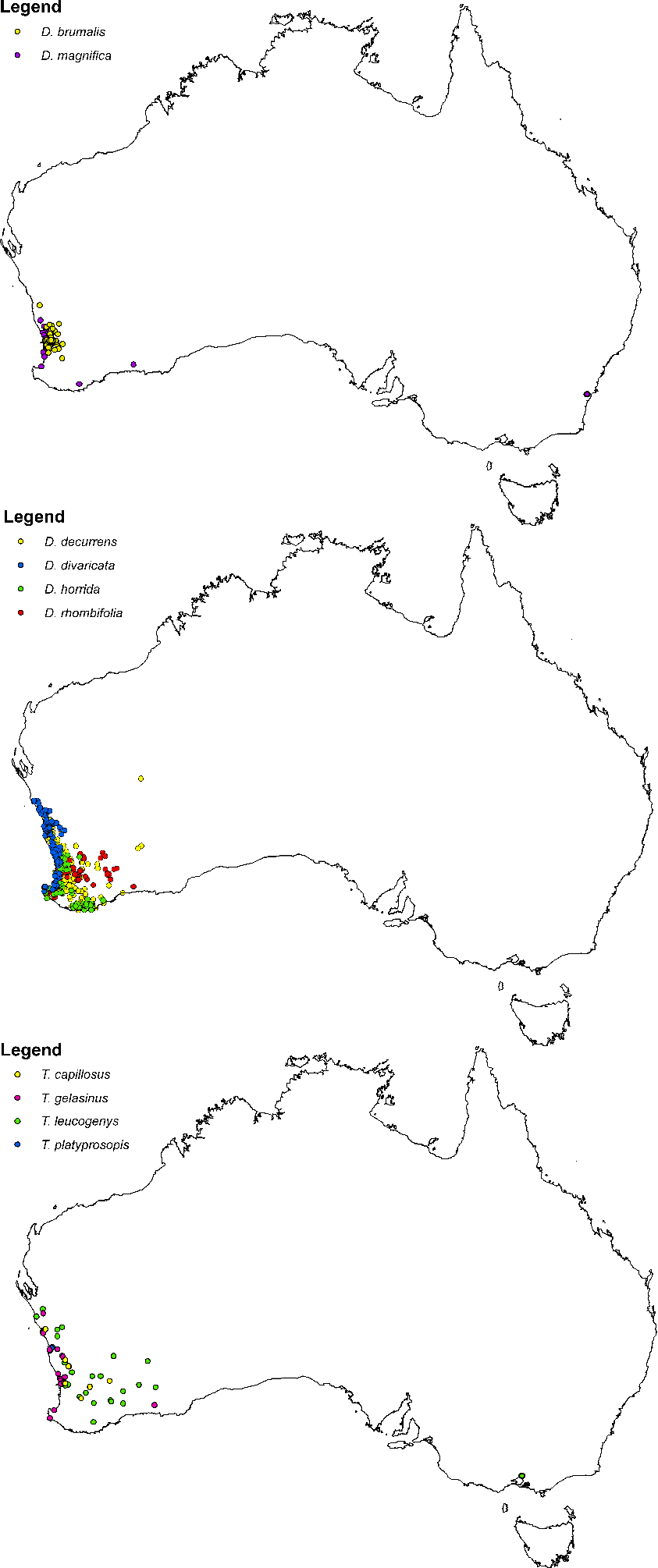


**Annex S3**. Records used in the ENM analyses.

| Species | decimalLongitude | decimalLatitude |
| --- | --- | --- |
| Daviesia_decurrens | 115.02833333 | -33.79055556 |
| Daviesia_decurrens | 115.066667 | -29.216667 |
| Daviesia_decurrens | 115.166667 | -30.066667 |
| Daviesia_decurrens | 115.21666667 | -34.24166667 |
| Daviesia_decurrens | 115.22472222 | -30.57666667 |
| Daviesia_decurrens | 115.283333 | -33.883333 |
| Daviesia_decurrens | 115.35 | -29.283333333 |
| Daviesia_decurrens | 115.48333333 | -30.76666667 |
| Daviesia_decurrens | 115.520405 | -33.892973 |
| Daviesia_decurrens | 115.53388889 | -29.71916667 |
| Daviesia_decurrens | 115.55 | -30.083333 |
| Daviesia_decurrens | 115.600278 | -33.449444 |
| Daviesia_decurrens | 115.635 | -32.63666667 |
| Daviesia_decurrens | 115.68333333 | -31.52166667 |
| Daviesia_decurrens | 115.716667 | -31.116667 |
| Daviesia_decurrens | 115.754387 | -33.97103 |
| Daviesia_decurrens | 115.816667 | -30.55 |
| Daviesia_decurrens | 115.85305556 | -30.81388889 |
| Daviesia_decurrens | 115.858611 | -31.955556 |
| Daviesia_decurrens | 115.8667 | -32.6167 |
| Daviesia_decurrens | 115.9667 | -32.3667 |
| Daviesia_decurrens | 115.97 | -33.52 |
| Daviesia_decurrens | 116.05 | -30.116667 |
| Daviesia_decurrens | 116.0667 | -31.3667 |
| Daviesia_decurrens | 116.12 | -33.97 |
| Daviesia_decurrens | 116.131389 | -31.978056 |
| Daviesia_decurrens | 116.15 | -33.15 |
| Daviesia_decurrens | 116.165659 | -32.625728 |
| Daviesia_decurrens | 116.216667 | -30.966667 |
| Daviesia_decurrens | 116.311944 | -33.387778 |
| Daviesia_decurrens | 116.38333333 | -33.83333333 |
| Daviesia_decurrens | 116.45 | -32.583333333 |
| Daviesia_decurrens | 116.483333 | -32.966667 |
| Daviesia_decurrens | 116.55 | -32.283333 |
| Daviesia_decurrens | 116.571667 | -31.73 |
| Daviesia_decurrens | 116.583333 | -31.966667 |
| Daviesia_decurrens | 116.64277778 | -34.34861111 |
| Daviesia_decurrens | 116.742778 | -33.957778 |
| Daviesia_decurrens | 116.75 | -33.333333333 |
| Daviesia_decurrens | 116.78638889 | -33.60138889 |
| Daviesia_decurrens | 116.855906667 | -33.016435 |
| Daviesia_decurrens | 116.8875 | -34.821389 |
| Daviesia_decurrens | 116.91666667 | -32.78305556 |
| Daviesia_decurrens | 116.983333 | -30.966667 |
| Daviesia_decurrens | 117.116667 | -34.533333 |
| Daviesia_decurrens | 117.15 | -33.833333333 |
| Daviesia_decurrens | 117.156827 | -35.032512 |
| Daviesia_decurrens | 117.18 | -32.93805556 |
| Daviesia_decurrens | 117.22 | -31.65 |
| Daviesia_decurrens | 117.324968 | -32.5398247 |
| Daviesia_decurrens | 117.366667 | -32.016667 |
| Daviesia_decurrens | 117.3667 | -34.25 |
| Daviesia_decurrens | 117.48166667 | -31.64472222 |
| Daviesia_decurrens | 117.5125 | -34.76472222 |
| Daviesia_decurrens | 117.55 | -33.683333 |
| Daviesia_decurrens | 117.6167 | -34.0167 |
| Daviesia_decurrens | 117.665 | -34.31166667 |
| Daviesia_decurrens | 117.751435 | -32.107809 |
| Daviesia_decurrens | 117.893592 | -34.6235317 |
| Daviesia_decurrens | 118.125253 | -34.337615 |
| Daviesia_decurrens | 118.26111111 | -34.89666667 |
| Daviesia_decurrens | 118.35 | -32.116667 |
| Daviesia_decurrens | 118.375278 | -32.375 |
| Daviesia_decurrens | 118.5783 | -34.2253 |
| Daviesia_decurrens | 118.781667 | -34.521667 |
| Daviesia_decurrens | 119.080323 | -33.474815 |
| Daviesia_decurrens | 119.978251753 | -33.9495384233 |
| Daviesia_decurrens | 120.9667 | -31.1167 |
| Daviesia_decurrens | 121.11528 | -26.68972 |
| Daviesia_divaricata | 114.35 | -28.066667 |
| Daviesia_divaricata | 114.631944 | -28.594444 |
| Daviesia_divaricata | 114.962143 | -29.700989 |
| Daviesia_divaricata | 114.988415 | -33.854717 |
| Daviesia_divaricata | 115.015212 | -28.734867 |
| Daviesia_divaricata | 115.023711 | -33.537638 |
| Daviesia_divaricata | 115.059248 | -28.989651 |
| Daviesia_divaricata | 115.079651 | -29.405647 |
| Daviesia_divaricata | 115.079651 | -30.27528 |
| Daviesia_divaricata | 115.187687 | -29.850504 |
| Daviesia_divaricata | 115.332868 | -28.784969 |
| Daviesia_divaricata | 115.397957 | -30.225168 |
| Daviesia_divaricata | 115.466213 | -30.664118 |
| Daviesia_divaricata | 115.520693 | -33.716768 |
| Daviesia_divaricata | 115.567506 | -29.563248 |
| Daviesia_divaricata | 115.622505 | -33.380372 |
| Daviesia_divaricata | 115.682309 | -31.5085 |
| Daviesia_divaricata | 115.700384 | -32.675457 |
| Daviesia_divaricata | 115.772311 | -33.06487 |
| Daviesia_divaricata | 115.789394 | -31.235259 |
| Daviesia_divaricata | 115.847772 | -32.223843 |
| Daviesia_divaricata | 115.869752 | -31.910302 |
| Daviesia_divaricata | 115.943107 | -32.720926 |
| Daviesia_divaricata | 115.988479 | -31.474806 |
| Daviesia_divaricata | 116.015556 | -29.881944 |
| Daviesia_divaricata | 116.066667 | -30.233333 |
| Daviesia_divaricata | 116.2894 | -29.9828 |
| Daviesia_horrida | 115.066667 | -33.566667 |
| Daviesia_horrida | 115.09861111 | -33.94027778 |
| Daviesia_horrida | 115.617583 | -34.072329 |
| Daviesia_horrida | 115.908828333 | -32.94067 |
| Daviesia_horrida | 115.925177778 | -33.2839 |
| Daviesia_horrida | 115.976577 | -32.657714 |
| Daviesia_horrida | 116.05 | -32.333333333 |
| Daviesia_horrida | 116.0667 | -31.3667 |
| Daviesia_horrida | 116.07 | -31.92 |
| Daviesia_horrida | 116.116667 | -33.533333 |
| Daviesia_horrida | 116.133333 | -33.966667 |
| Daviesia_horrida | 116.17 | -31.67 |
| Daviesia_horrida | 116.294986 | -32.035715 |
| Daviesia_horrida | 116.47 | -31.55 |
| Daviesia_horrida | 116.56386111 | -32.43797222 |
| Daviesia_horrida | 116.757904 | -33.960181 |
| Daviesia_horrida | 116.83333333 | -34.86666667 |
| Daviesia_horrida | 117.020693 | -34.627129 |
| Daviesia_horrida | 117.22 | -31.65 |
| Daviesia_horrida | 117.228333 | -34.854444 |
| Daviesia_horrida | 117.525 | -35.015 |
| Daviesia_horrida | 117.65 | -34.65 |
| Daviesia_horrida | 117.73166667 | -34.33805556 |
| Daviesia_horrida | 117.944922676 | -34.9799631699 |
| Daviesia_horrida | 118.05 | -34.65 |
| Daviesia_horrida | 118.75 | -34.433333 |
| Daviesia_rhombifolia | 115.09805556 | -33.60305556 |
| Daviesia_rhombifolia | 115.25 | -30.23 |
| Daviesia_rhombifolia | 115.266667 | -33.95 |
| Daviesia_rhombifolia | 115.552778 | -34.013889 |
| Daviesia_rhombifolia | 115.733333 | -33.483333 |
| Daviesia_rhombifolia | 115.92305556 | -33.15805556 |
| Daviesia_rhombifolia | 115.97 | -32.42 |
| Daviesia_rhombifolia | 116.05833333 | -31.96138889 |
| Daviesia_rhombifolia | 116.06472222 | -32.715 |
| Daviesia_rhombifolia | 116.293033 | -32.040443 |
| Daviesia_rhombifolia | 116.37 | -31.77 |
| Daviesia_rhombifolia | 116.45 | -32.583333 |
| Daviesia_rhombifolia | 116.45 | -32.95 |
| Daviesia_rhombifolia | 116.601667 | -31.885833 |
| Daviesia_rhombifolia | 116.85 | -32.483333 |
| Daviesia_rhombifolia | 116.916313333 | -32.7564416667 |
| Daviesia_rhombifolia | 117.073994 | -33.126305 |
| Daviesia_rhombifolia | 117.31666667 | -32.53333333 |
| Daviesia_rhombifolia | 117.48166667 | -31.64472222 |
| Daviesia_rhombifolia | 117.694505 | -33.077494 |
| Daviesia_rhombifolia | 118.116944 | -33.45 |
| Daviesia_rhombifolia | 118.54972222 | -31.75 |
| Daviesia_rhombifolia | 118.68333333 | -31.38305556 |
| Daviesia_rhombifolia | 118.87 | -32.45166667 |
| Daviesia_rhombifolia | 119.016667 | -32.766667 |
| Daviesia_rhombifolia | 119.214722 | -33.095278 |
| Daviesia_rhombifolia | 119.605 | -32.38666667 |
| Daviesia_rhombifolia | 120.636667 | -33.543889 |
| Diuris_brumalis | 115.4153 | -29.6661 |
| Diuris_brumalis | 115.783569 | -31.914982 |
| Diuris_brumalis | 115.86138889 | -31.02861111 |
| Diuris_brumalis | 115.965 | -32.64972222 |
| Diuris_brumalis | 116.00472222 | -32.18666667 |
| Diuris_brumalis | 116.008089 | -32.41075 |
| Diuris_brumalis | 116.01666667 | -31.88333333 |
| Diuris_brumalis | 116.113361 | -31.486972 |
| Diuris_brumalis | 116.195556 | -30.953056 |
| Diuris_brumalis | 116.35919 | -32.4946166667 |
| Diuris_brumalis | 116.3975 | -31.31916667 |
| Diuris_brumalis | 116.453629265 | -31.8822912311 |
| Diuris_brumalis | 116.483333333 | -32.116666667 |
| Diuris_brumalis | 116.619266 | -32.402665 |
| Diuris_brumalis | 116.638333 | -30.837222 |
| Diuris_brumalis | 116.855295344 | -33.0161942576 |
| Diuris_brumalis | 116.873161 | -32.101479 |
| Diuris_magnifica | 115.466471 | -30.608377 |
| Diuris_magnifica | 115.51401 | -33.538802 |
| Diuris_magnifica | 115.643188 | -32.642467 |
| Diuris_magnifica | 115.702988 | -30.982371 |
| Diuris_magnifica | 115.711944 | -32.916944 |
| Diuris_magnifica | 115.722865 | -31.56149 |
| Diuris_magnifica | 115.81968 | -31.835537 |
| Diuris_magnifica | 115.824963 | -32.371809 |
| Diuris_magnifica | 115.830208 | -32.081203 |
| Diuris_magnifica | 116.048194 | -31.327333 |
| Diuris_magnifica | 117.933137 | -34.651112 |
| Diuris_magnifica | 121.373333 | -33.402222 |
| Diuris_magnifica | 150.199444 | -35.291389 |
| Trichocolletes_capillosus | 114.791062964 | -28.4985895352 |
| Trichocolletes_capillosus | 116.0364 | -30.4638 |
| Trichocolletes_capillosus | 116.048187366 | -31.9341153414 |
| Trichocolletes_capillosus | 116.256268549 | -30.8621688416 |
| Trichocolletes_capillosus | 117.04249 | -32.8761 |
| Trichocolletes_capillosus | 117.614920404 | -32.144756614 |
| Trichocolletes_capillosus | 118.7586 | 31.1981 |
| Trichocolletes_capillosus | 118.8731 | -31.7939 |
| Trichocolletes_gelasinus | 114.614892341 | -28.6725061123 |
| Trichocolletes_gelasinus | 114.636207587 | -27.4946676636 |
| Trichocolletes_gelasinus | 115.057598443 | -29.8540143475 |
| Trichocolletes_gelasinus | 115.059813018 | -34.1352092077 |
| Trichocolletes_gelasinus | 115.344656627 | -33.6516630792 |
| Trichocolletes_gelasinus | 115.526930824 | -29.7832201216 |
| Trichocolletes_gelasinus | 115.596467805 | -31.3437016937 |
| Trichocolletes_gelasinus | 115.7067 | -31.6294 |
| Trichocolletes_gelasinus | 115.77 | -31.74 |
| Trichocolletes_gelasinus | 115.833074697 | -31.9606734731 |
| Trichocolletes_gelasinus | 115.878873296 | -30.2451964979 |
| Trichocolletes_gelasinus | 115.934231069 | -31.8690345293 |
| Trichocolletes_gelasinus | 116.022599062 | -31.5292322769 |
| Trichocolletes_gelasinus | 121.717495667 | -33.3299220336 |
| Trichocolletes_leucogenys | 0.0 | -32.98 |
| Trichocolletes_leucogenys | 114.210756484 | -27.6956038264 |
| Trichocolletes_leucogenys | 114.614499339 | -27.2127343035 |
| Trichocolletes_leucogenys | 114.631056844 | -28.6134084163 |
| Trichocolletes_leucogenys | 115.348206721 | -33.6515557274 |
| Trichocolletes_leucogenys | 115.525776046 | -28.5230268396 |
| Trichocolletes_leucogenys | 115.548633 | -28.955781 |
| Trichocolletes_leucogenys | 115.765201322 | -31.9708761673 |
| Trichocolletes_leucogenys | 115.852674046 | -28.3770041289 |
| Trichocolletes_leucogenys | 116.017419707 | -30.6403246893 |
| Trichocolletes_leucogenys | 116.041947277 | -32.0650638216 |
| Trichocolletes_leucogenys | 116.061355464 | -31.9183253206 |
| Trichocolletes_leucogenys | 116.208760404 | -32.0158212895 |
| Trichocolletes_leucogenys | 116.263480422 | -30.8465479633 |
| Trichocolletes_leucogenys | 116.475821122 | -31.2409325215 |
| Trichocolletes_leucogenys | 116.9675 | -32.7802 |
| Trichocolletes_leucogenys | 117.04249 | -32.8761 |
| Trichocolletes_leucogenys | 117.336575961 | -32.5531263883 |
| Trichocolletes_leucogenys | 117.615263727 | -32.1435938321 |
| Trichocolletes_leucogenys | 117.741691317 | -31.4892911354 |
| Trichocolletes_leucogenys | 117.833333 | -34.383333 |
| Trichocolletes_leucogenys | 117.8486 | -33.216 |
| Trichocolletes_leucogenys | 117.8486 | 33.22 |
| Trichocolletes_leucogenys | 118.274425939 | -31.4872618872 |
| Trichocolletes_leucogenys | 118.901606349 | -33.0035291522 |
| Trichocolletes_leucogenys | 118.9533 | 31.7847 |
| Trichocolletes_leucogenys | 118.96388 | -33.10111 |
| Trichocolletes_leucogenys | 119.120342571 | -30.2071614975 |
| Trichocolletes_leucogenys | 119.707170437 | -32.430683468 |
| Trichocolletes_leucogenys | 119.71472 | -33.22166 |
| Trichocolletes_leucogenys | 120.714881322 | -32.0372271049 |
| Trichocolletes_leucogenys | 121.265729294 | -30.526524678 |
| Trichocolletes_leucogenys | 121.790323823 | -32.1999580689 |
| Trichocolletes_leucogenys | 145.0681094 | -37.8123054133 |
| Trichocolletes_platyprosopis | 114.628315219 | -28.5879423175 |
| Trichocolletes_platyprosopis | 114.7986071 | -28.4974827833 |
| Trichocolletes_platyprosopis | 115.1519 | -29.7197 |
| Trichocolletes_platyprosopis | 115.83 | -30.2 |

**Annex S4**. Pearsons' correlation coefficient computed for 19 bioclimatic variables.

|  | bio1 | bio2 | bio3 | bio4 | bio5 | bio6 | bio7 | bio8 | bio9 | bio10 | bio11 | bio12 | bio13 | bio14 | bio15 | bio16 | bio17 | bio18 | bio19 |
| --- | --- | --- | --- | --- | --- | --- | --- | --- | --- | --- | --- | --- | --- | --- | --- | --- | --- | --- | --- |
| bio1 | x | 0,356 | 0,397 | -0,146 | 0,830 | 0,813 | 0,093 | 0,817 | 0,485 | 0,919 | 0,936 | 0,023 | 0,404 | -0,792 | 0,831 | 0,365 | -0,775 | 0,384 | -0,730 |
| bio2 | x | x | -0,096 | 0,579 | 0,704 | -0,154 | 0,819 | 0,431 | 0,007 | 0,563 | 0,114 | -0,688 | -0,479 | -0,510 | 0,056 | -0,516 | -0,529 | -0,426 | -0,547 |
| bio3 | x | x | x | -0,846 | -0,023 | 0,680 | -0,621 | 0,094 | 0,446 | 0,065 | 0,641 | 0,506 | 0,682 | -0,274 | 0,622 | 0,682 | -0,264 | 0,550 | -0,185 |
| bio4 | x | x | x | x | 0,382 | -0,633 | 0,927 | 0,139 | -0,352 | 0,238 | -0,472 | -0,788 | -0,820 | -0,053 | -0,501 | -0,837 | -0,072 | -0,679 | -0,140 |
| bio5 | x | x | x | x | x | 0,417 | 0,601 | 0,765 | 0,341 | 0,964 | 0,612 | -0,444 | -0,089 | -0,817 | 0,490 | -0,132 | -0,817 | -0,083 | -0,756 |
| bio6 | x | x | x | x | x | x | -0,468 | 0,488 | 0,629 | 0,564 | 0,947 | 0,443 | 0,742 | -0,576 | 0,871 | 0,726 | -0,552 | 0,629 | -0,422 |
| bio7 | x | x | x | x | x | x | x | 0,315 | -0,222 | 0,442 | -0,236 | -0,819 | -0,737 | -0,288 | -0,286 | -0,765 | -0,309 | -0,632 | -0,363 |
| bio8 | x | x | x | x | x | x | x | x | 0,027 | 0,845 | 0,669 | -0,104 | 0,184 | -0,590 | 0,561 | 0,142 | -0,578 | 0,295 | -0,719 |
| bio9 | x | x | x | x | x | x | x | x | x | 0,358 | 0,572 | 0,094 | 0,320 | -0,513 | 0,543 | 0,310 | -0,499 | 0,132 | -0,169 |
| bio10 | x | x | x | x | x | x | x | x | x | x | 0,739 | -0,280 | 0,084 | -0,806 | 0,615 | 0,040 | -0,798 | 0,107 | -0,769 |
| bio11 | x | x | x | x | x | x | x | x | x | x | x | 0,287 | 0,639 | -0,695 | 0,910 | 0,611 | -0,674 | 0,565 | -0,596 |
| bio12 | x | x | x | x | x | x | x | x | x | x | x | x | 0,893 | 0,373 | 0,371 | 0,914 | 0,406 | 0,823 | 0,364 |
| bio13 | x | x | x | x | x | x | x | x | x | x | x | x | x | -0,050 | 0,692 | 0,994 | -0,019 | 0,885 | 0,000 |
| bio14 | x | x | x | x | x | x | x | x | x | x | x | x | x | x | -0,592 | -0,017 | 0,992 | 0,045 | 0,771 |
| bio15 | x | x | x | x | x | x | x | x | x | x | x | x | x | x | x | 0,657 | -0,564 | 0,610 | -0,406 |
| bio16 | x | x | x | x | x | x | x | x | x | x | x | x | x | x | x | x | 0,013 | 0,877 | 0,034 |
| bio17 | x | x | x | x | x | x | x | x | x | x | x | x | x | x | x | x | x | 0,078 | 0,786 |
| bio18 | x | x | x | x | x | x | x | x | x | x | x | x | x | x | x | x | x | x | -0,081 |
| bio19 | x | x | x | x | x | x | x | x | x | x | x | x | x | x | x | x | x | x | x |

**Annex S5**. Bioclimatic variables. Layers used in ENM analyses marked with ‘+”.

| **Code** | **Description** | **ENM** |
| --- | --- | --- |
| bio1 | annual mean temperature | + |
| bio2 | mean diurnal range [mean of monthly (max temp - min temp)] | + |
| bio3 | isothermality (bio2/bio7) (×100) | + |
| bio4 | temperature seasonality (standard deviation ×100) |  |
| bio5 | max temperature of warmest month |  |
| bio6 | min temperature of coldest month |  |
| bio7 | temperature annual range (bio5-bio6) |  |
| bio8 | mean temperature of wettest quarter |  |
| bio9 | mean temperature of driest quarter | + |
| bio10 | mean temperature of warmest quarter |  |
| bio11 | mean temperature of coldest quarter |  |
| bio12 | annual precipitation | + |
| bio13 | precipitation of wettest month |  |
| bio14 | precipitation of driest month | + |
| bio15 | precipitation seasonality (coefficient of variation) |  |
| bio16 | precipitation of wettest quarter |  |
| bio17 | precipitation of driest quarter | + |
| bio18 | precipitation of warmest quarter |  |
| bio19 | precipitation of coldest quarter | + |

**Annex S6**. Modelling performance indexes.

| **Species** | **AUC (standard deviation)** | **TSS** | **Max Kappa** | **Specficity** |
| --- | --- | --- | --- | --- |
| *Diuris brumalis* | 0.997 (0.001) | 0.985 | 0.489 | 0.985 |
| *Diuris magnifica* | 0.980 (0.010) | 0.887 | 0.449 | 0.938 |
| *Daviesia decurrens* | 0.983 (0.003) | 0.922 | 0.474 | 0.937 |
| *Daviesia divaricata* | 0.996 (0.001) | 0.986 | 0.492 | 0.986 |
| *Daviesia horrida* | 0.994 (0.001) | 0.979 | 0.49 | 0.979 |
| *Daviesia rhombifolia* | 0.990 (0.002) | 0.937 | 0.479 | 0.937 |
| *Trichocolletes capillosus* | 0.991 (0.002) | 0.835 | 0.438 | 0.835 |
| *Trichocolletes gelasinus* | 0.987 (0.008) | 0.876 | 0.446 | 0.930 |
| *Trichocolletes leucogenys* | 0.975 (0.007) | 0.817 | 0.427 | 0.892 |
| *Trichocolletes platyprosopis* | 0.995 (0.003) | 0.943 | 0.456 | 0.943 |

**Annex S7**. Results of the jackknife test of variable importance.


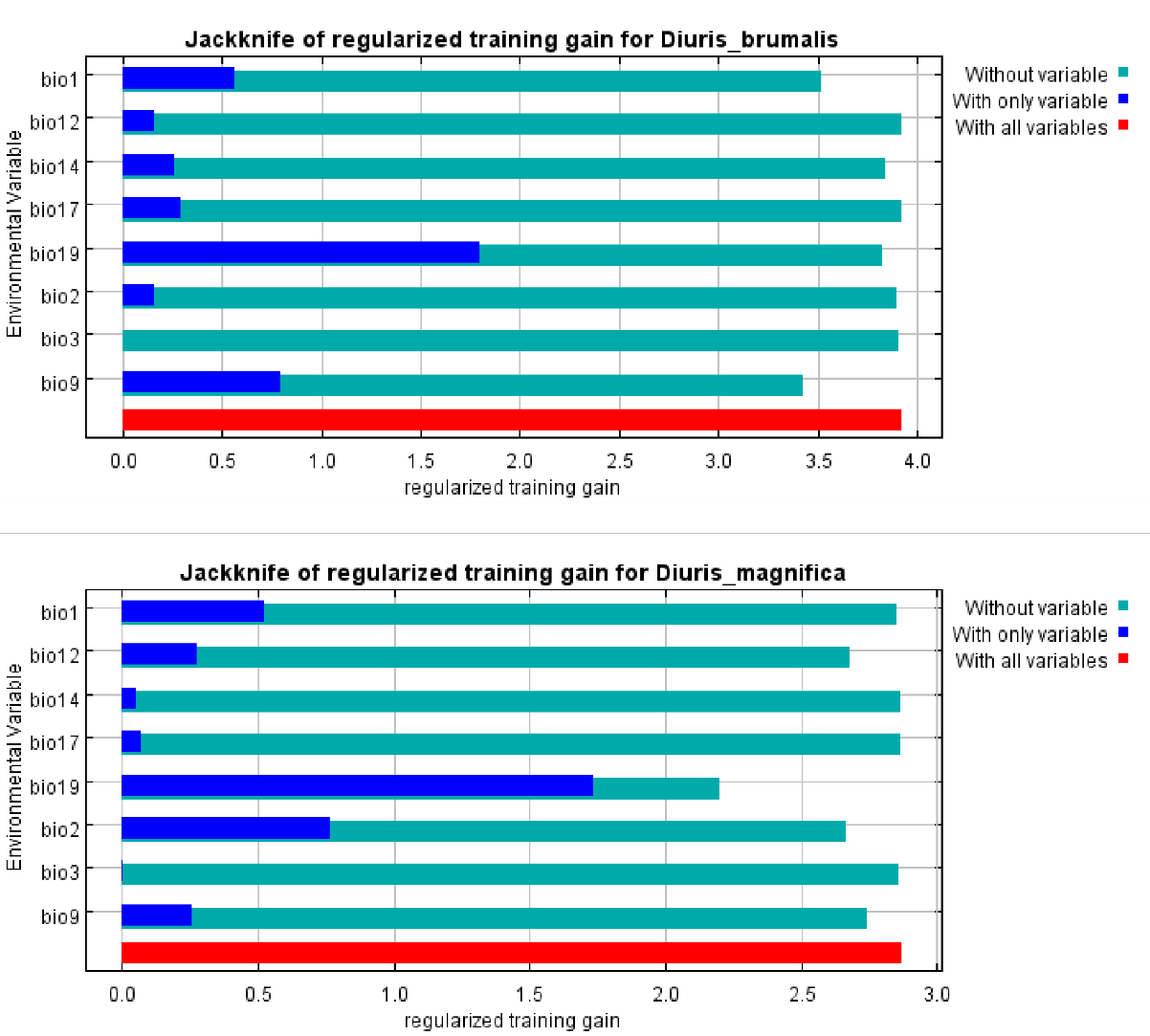


Figure 1. Results of the jackknife test of variable importance – orchid models.


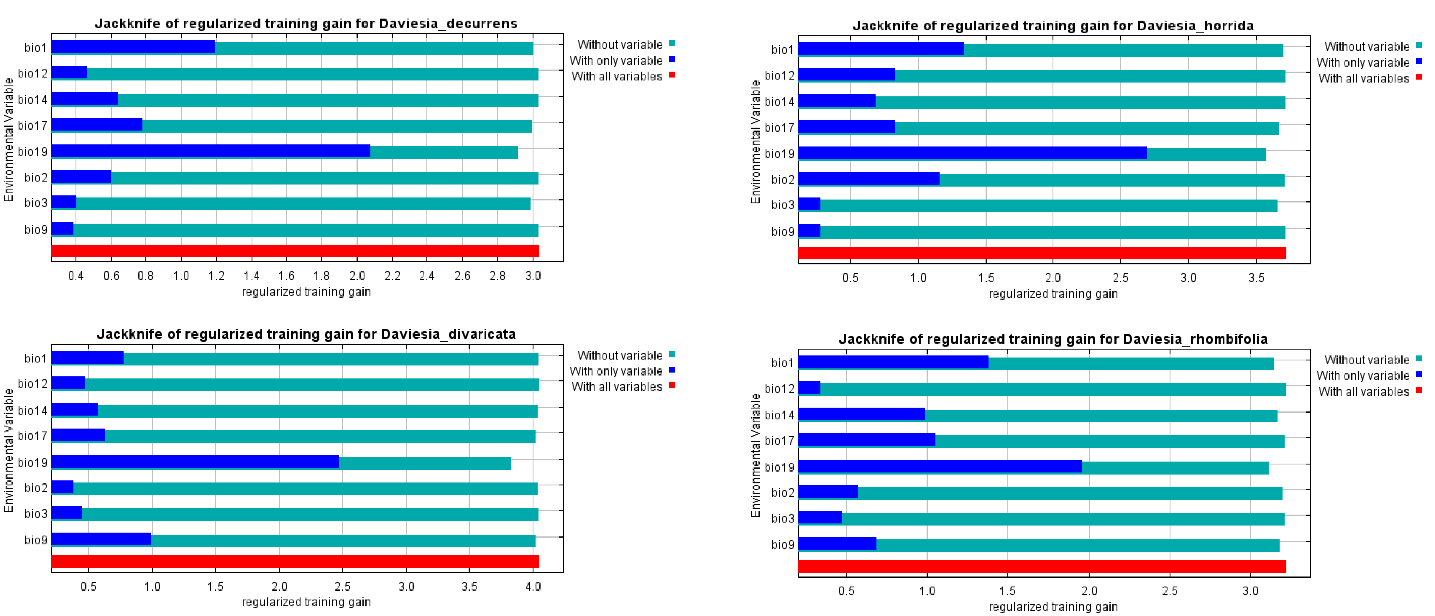


Figure 2. Results of the jackknife test of variable importance – *Daviesia* models.


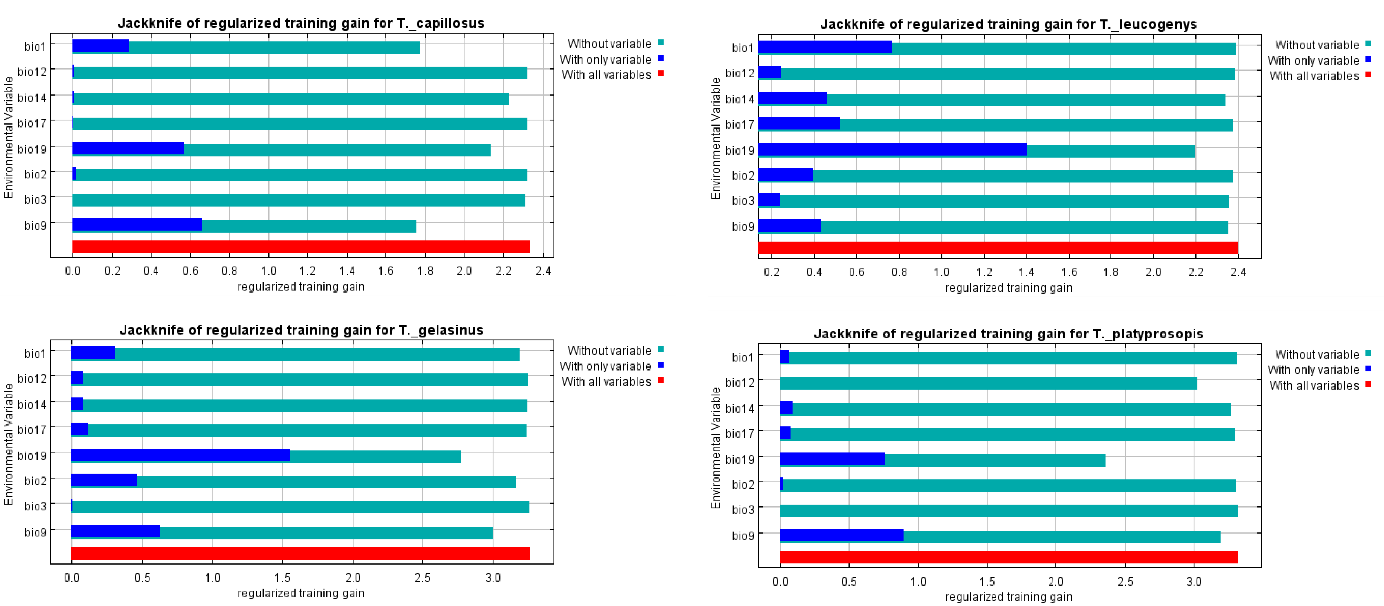


Figure 3. Results of the jackknife test of variable importance – pollinators models.

**Annex S8**. Predicted niche occupancy (PNO) profiles for the species studied.


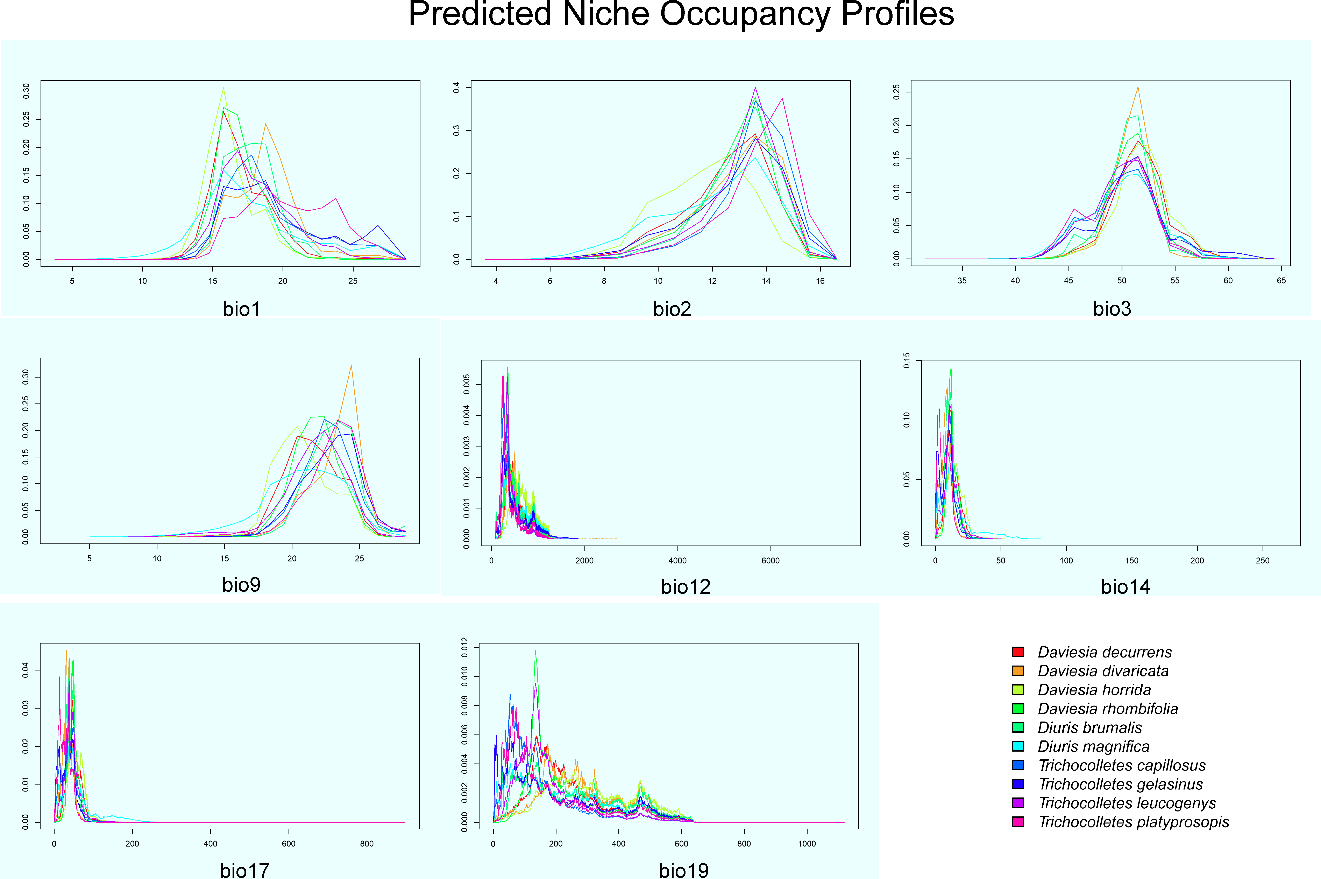


**Annex S9**. Changes in the distribution of suitable niches for *Daviesia* species studied.


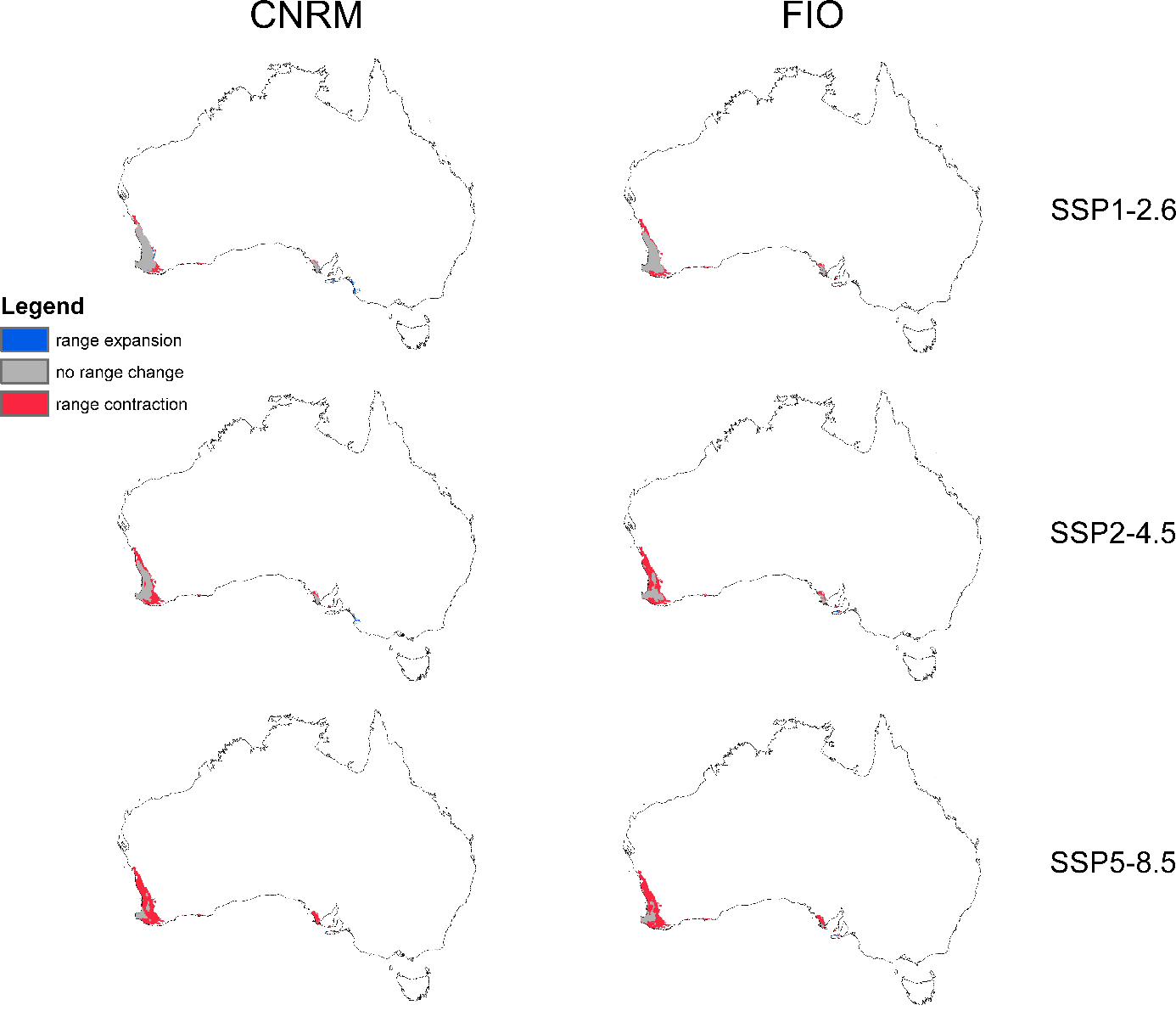


Figure 1. Changes in Daviesia decurrens distribution according to the CNRM and FIO projections and three various climate change scenarios.


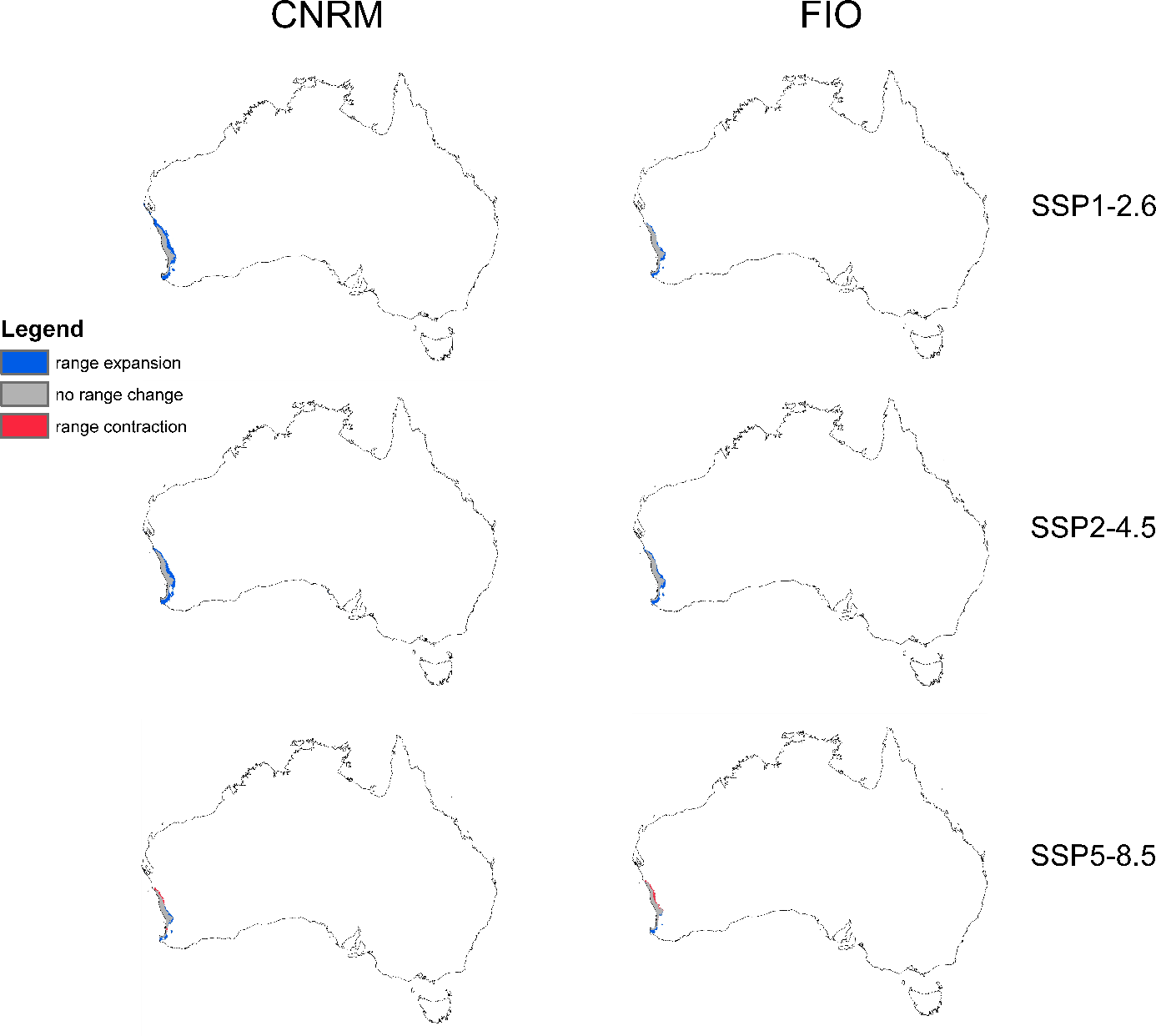


Figure 2. Changes in Daviesia divaricata distribution according to the CNRM and FIO projections and three various climate change scenarios.


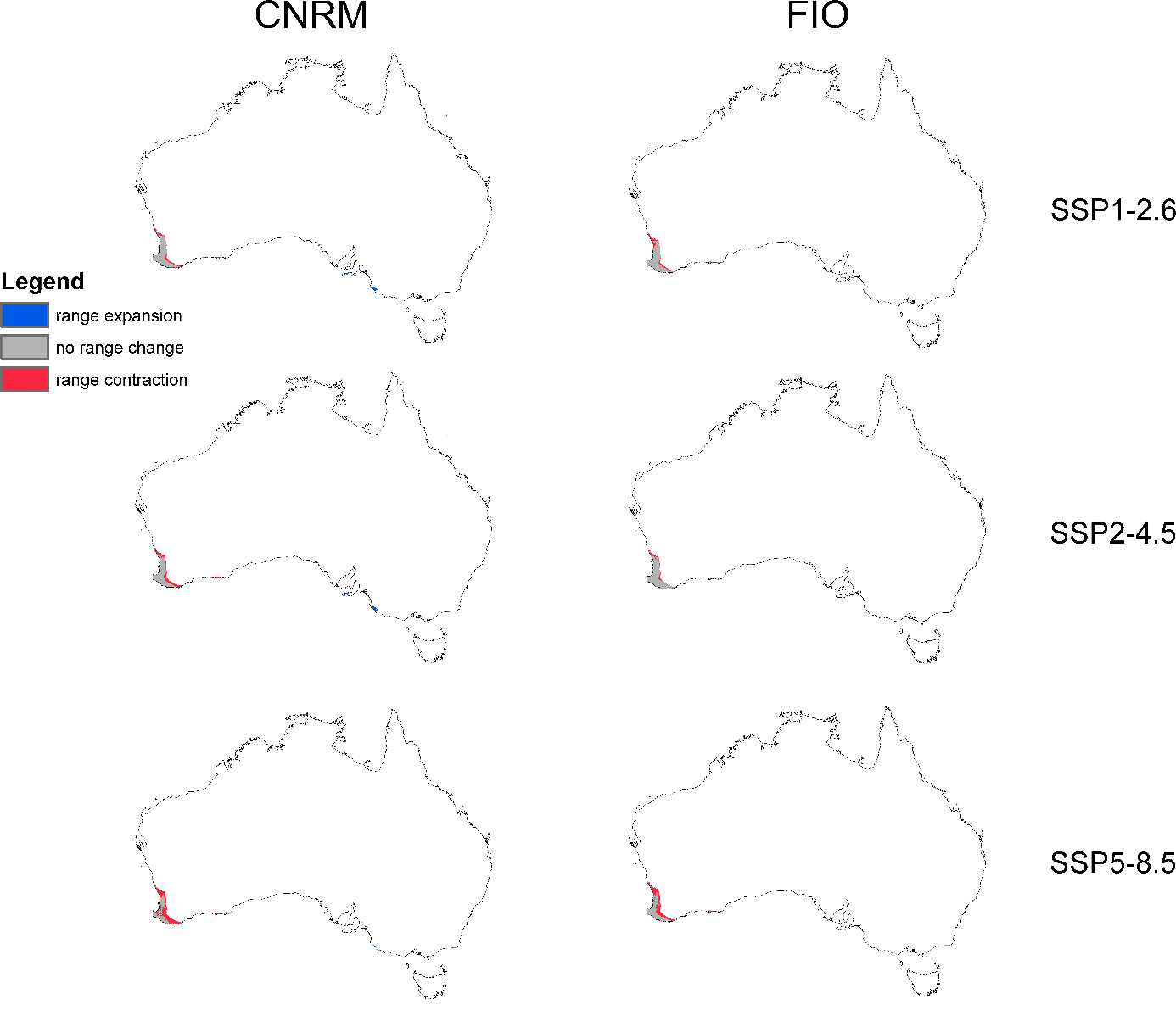


Figure 3. Changes in Daviesia horrida distribution according to the CNRM and FIO projections and three various climate change scenarios.


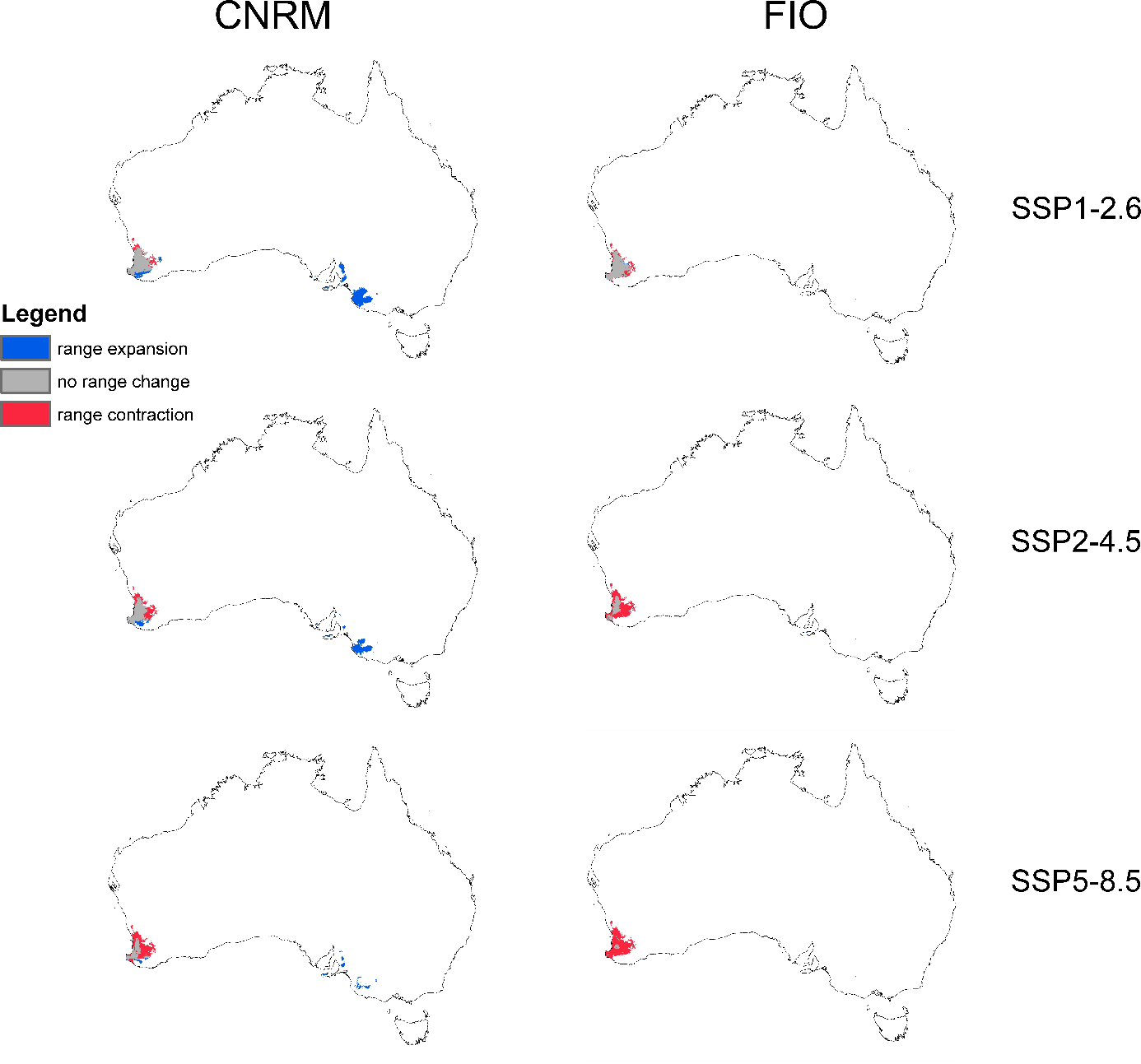


Figure 4. Changes in Daviesia rhombifolia distribution according to the CNRM and FIO projections and three various climate change scenarios.

**Annex S10**. Changes in the distribution of suitable niches for pollinators studied.


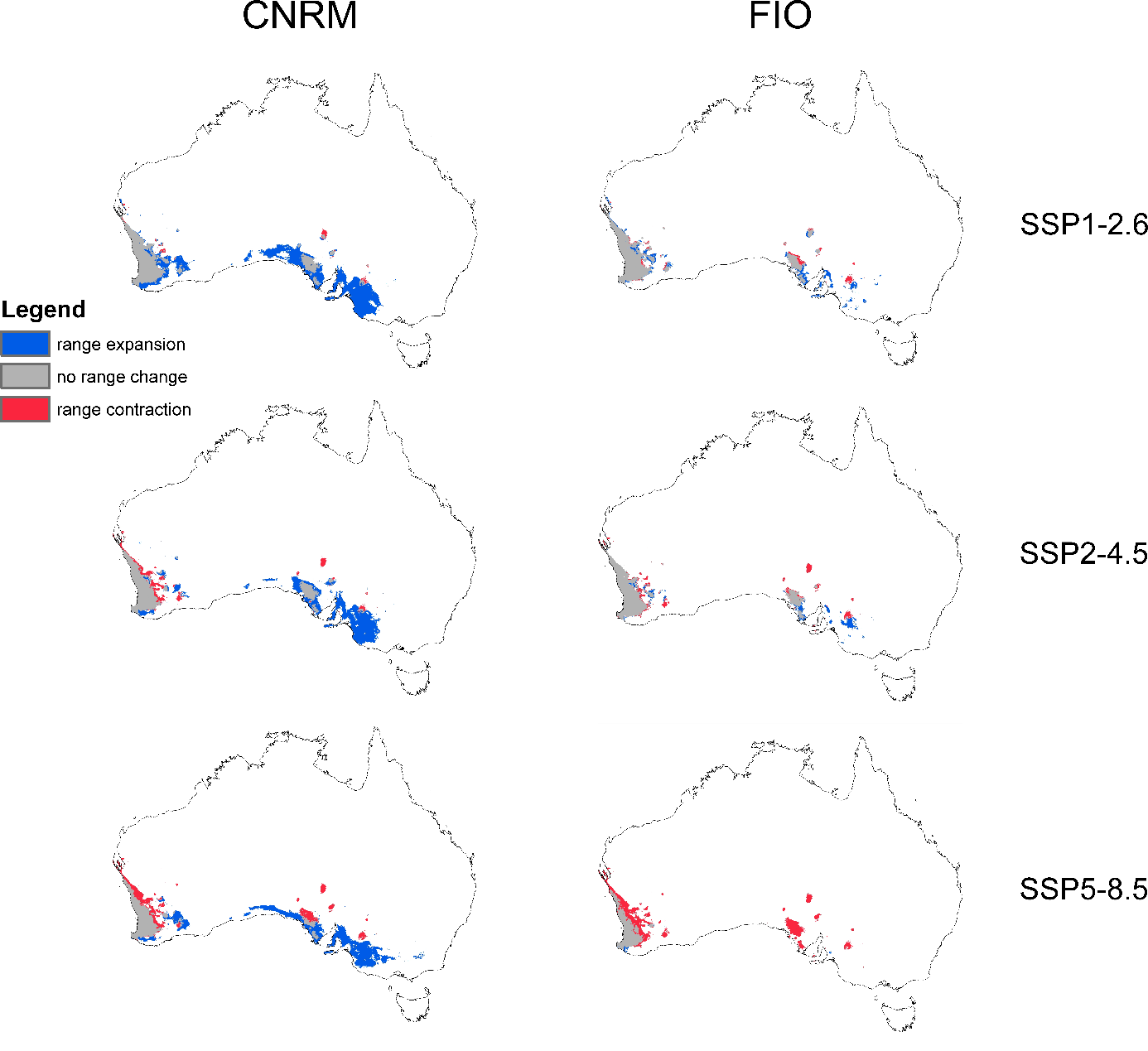


Figure 1. Changes in Trichocolletes capillosus distribution according to the CNRM and FIO projections and three various climate change scenarios.


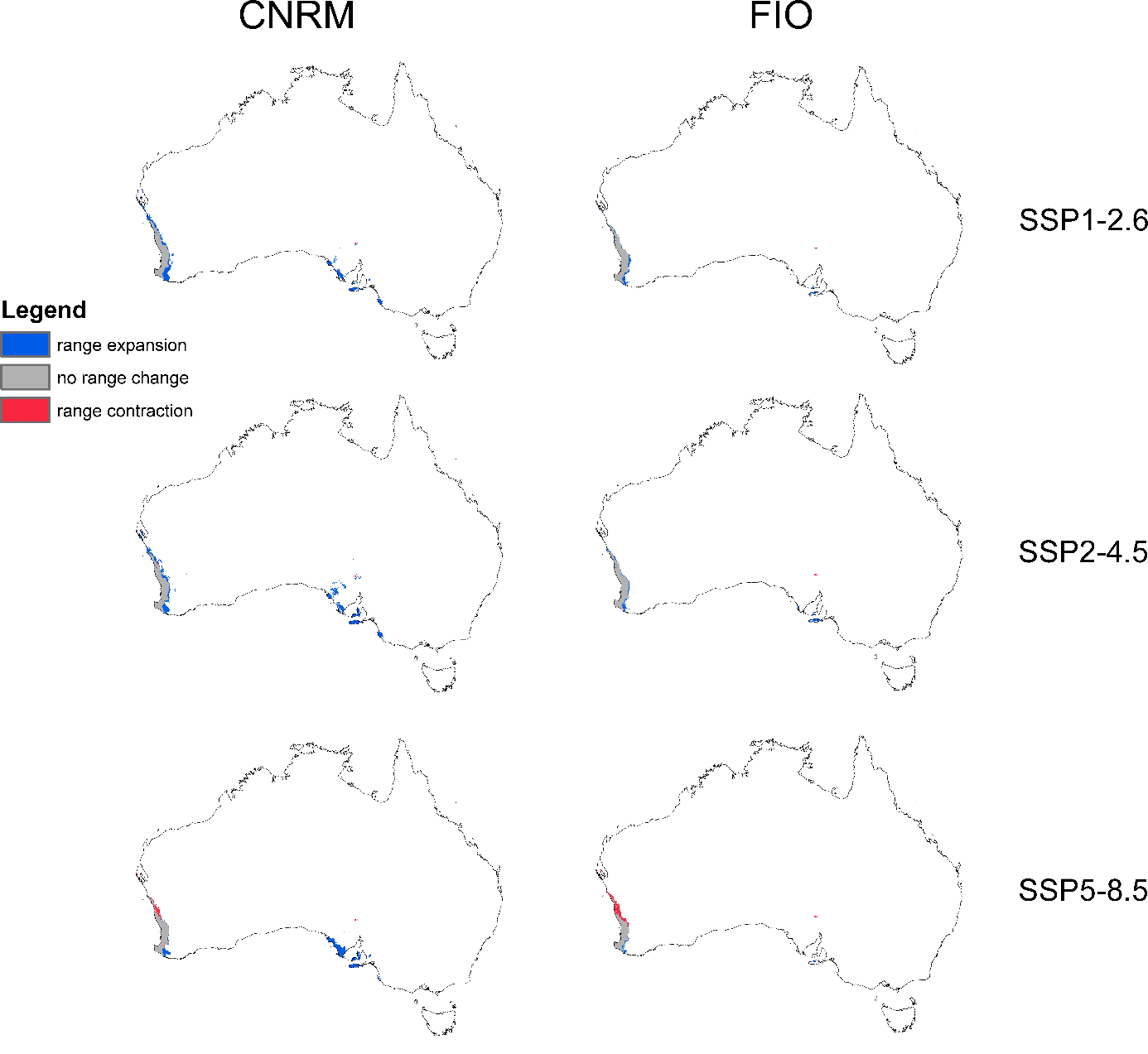


Figure 2. Changes in Trichocolletes gelasinus distribution according to the CNRM and FIO projections and three various climate change scenarios.


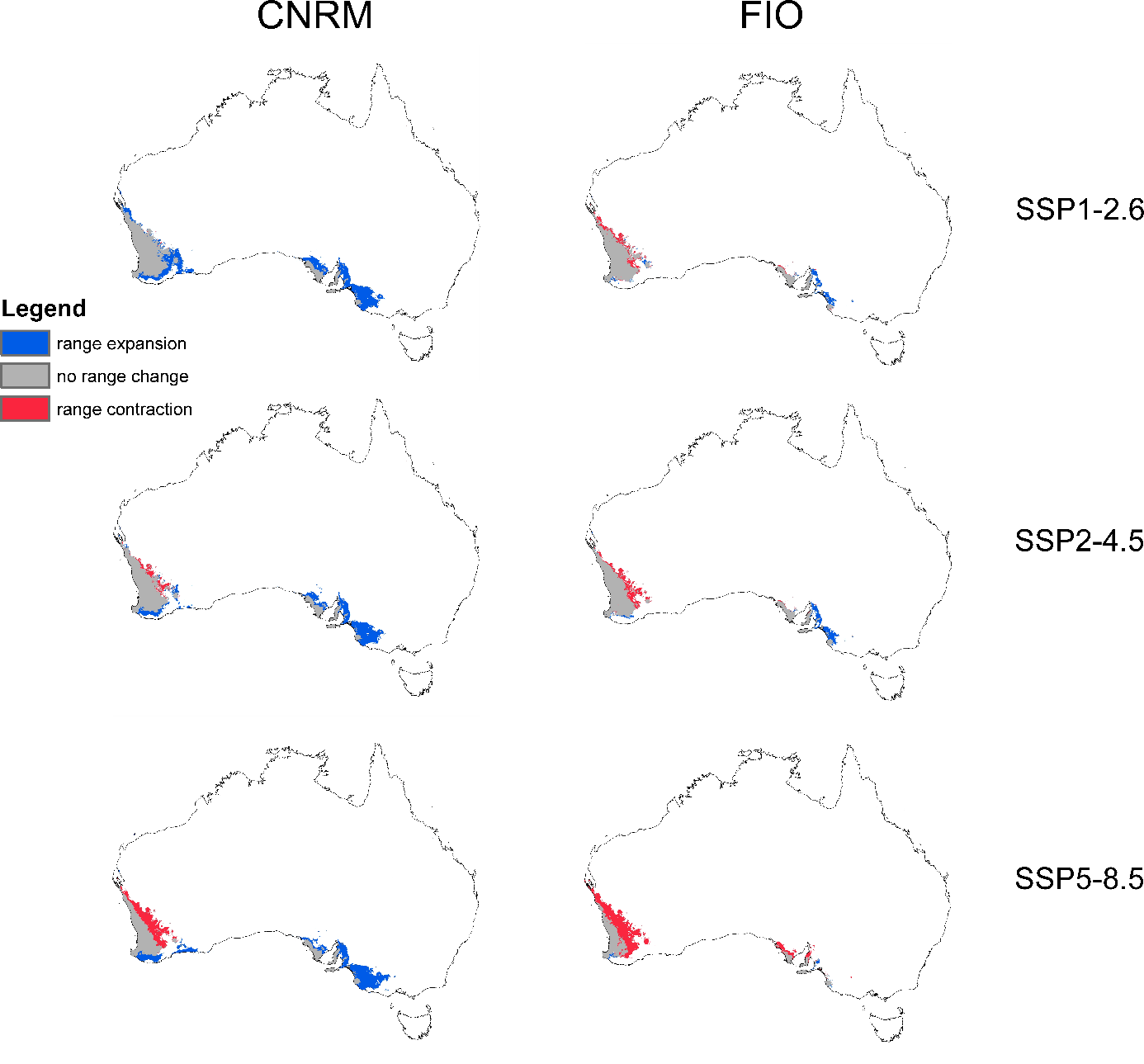


Figure 3. Changes in Trichocolletes leucogenys distribution according to the CNRM and FIO projections and three various climate change scenarios.


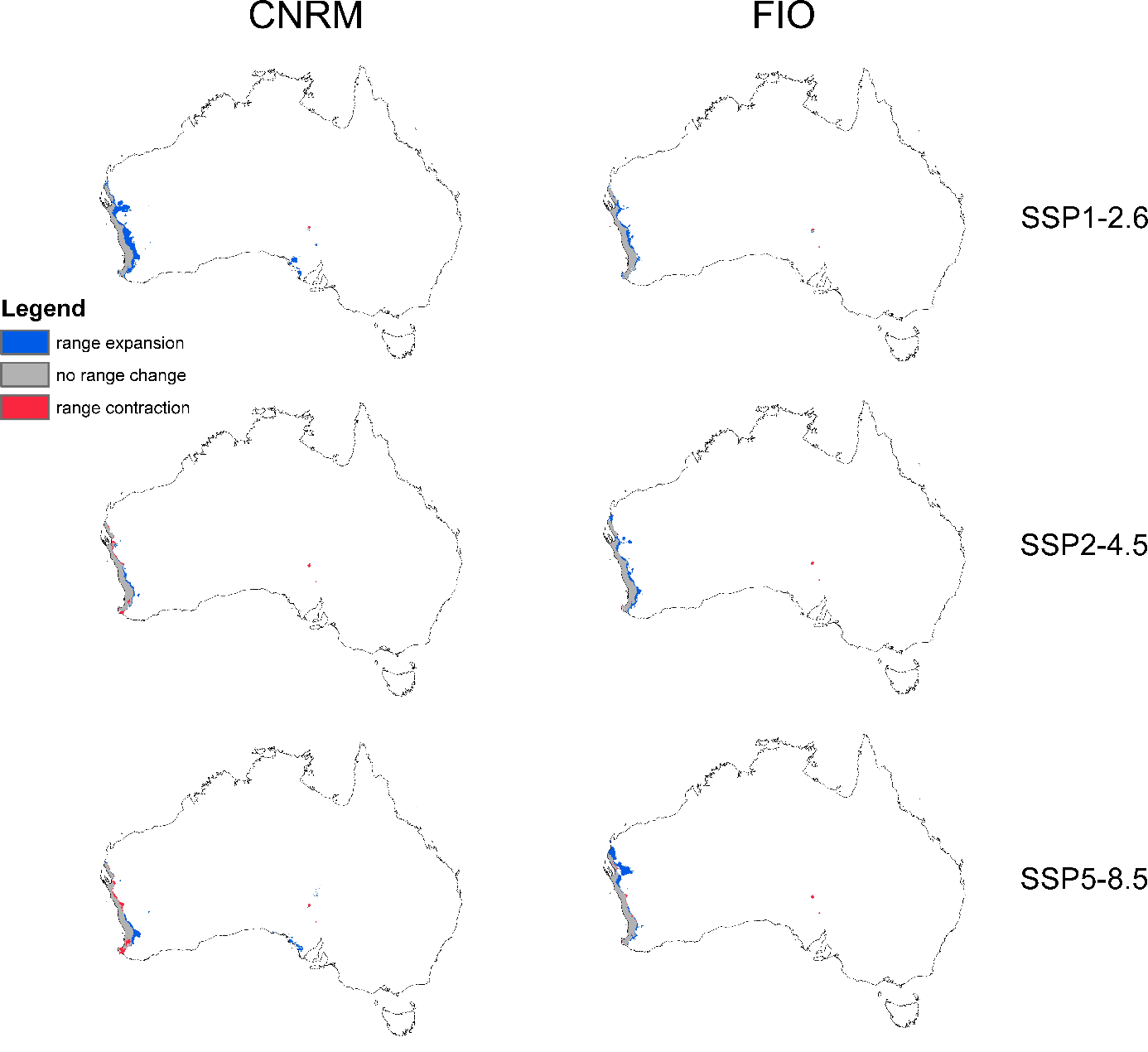


Figure 4. Changes in Trichocolletes platyprosopis distribution according to the CNRM and FIO projections and three various climate change scenarios.

**Annex S11**. Overlap of potential range of *Daviesia* representatives and *Diuris brumalis.*


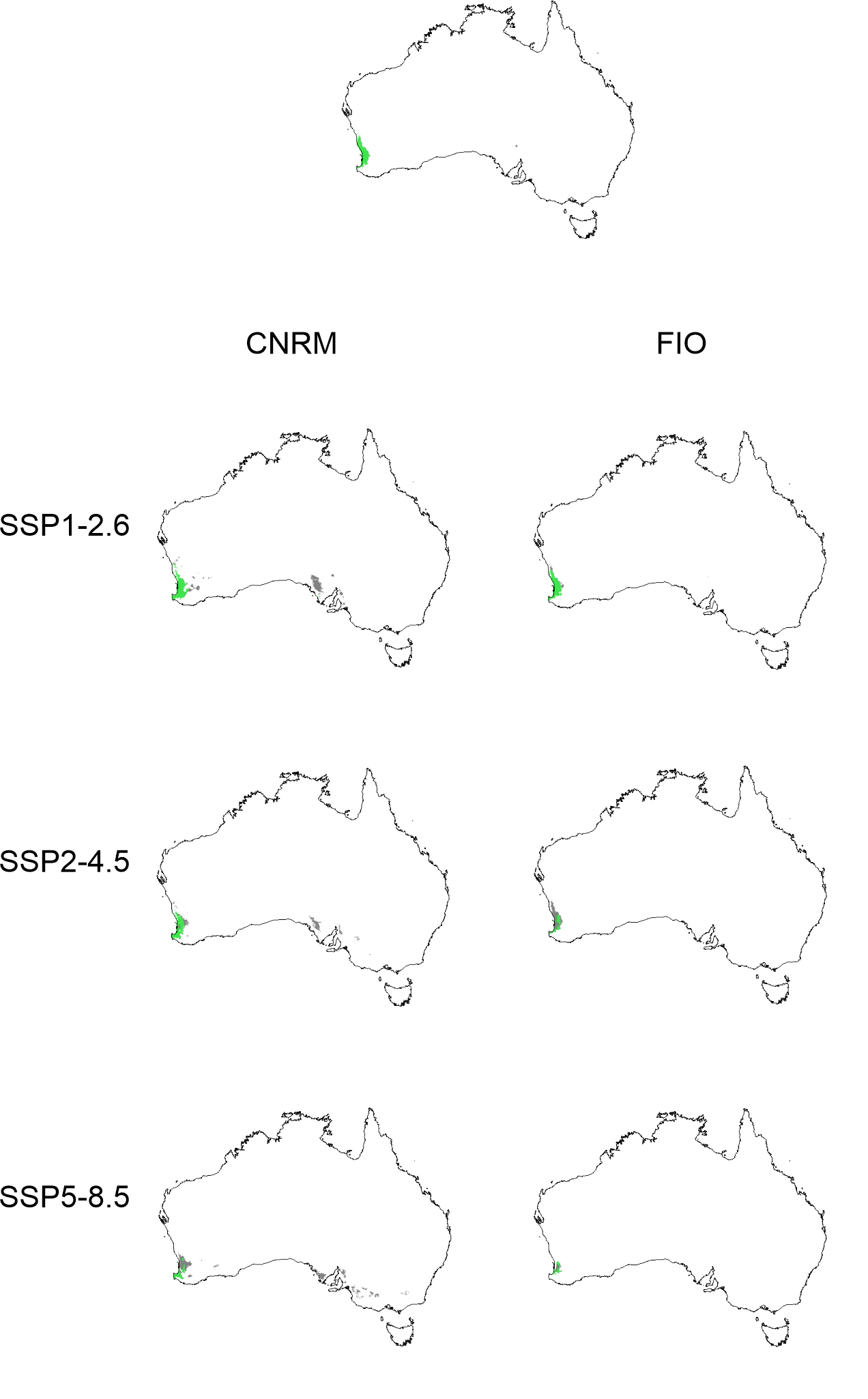


Figure 1. Overlap of potential range of Daviesia decurrens and Diuris brumalis. Areas of overlap marked with green, areas suitable only for orchid occurrence marked in grey. Upper map present current overlap between the species.


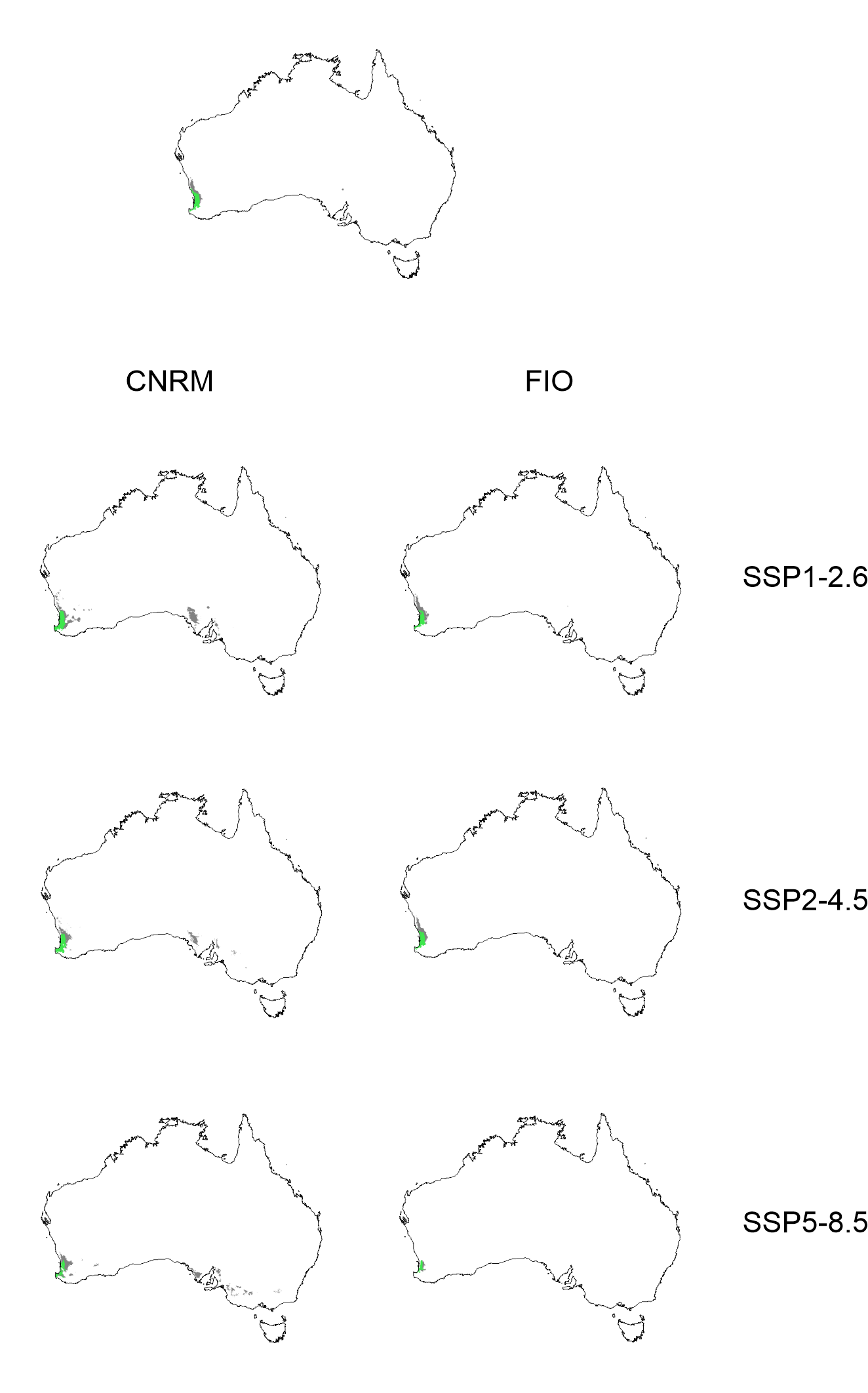


Figure 2. Overlap of potential range of Daviesia horrida and Diuris brumalis. Areas of overlap marked with green, areas suitable only for orchid occurrence marked in grey. Upper map present current overlap between the species.

**Annex S12**. Overlap of potential range of *Daviesia* representative and *Diuris magnifica.*


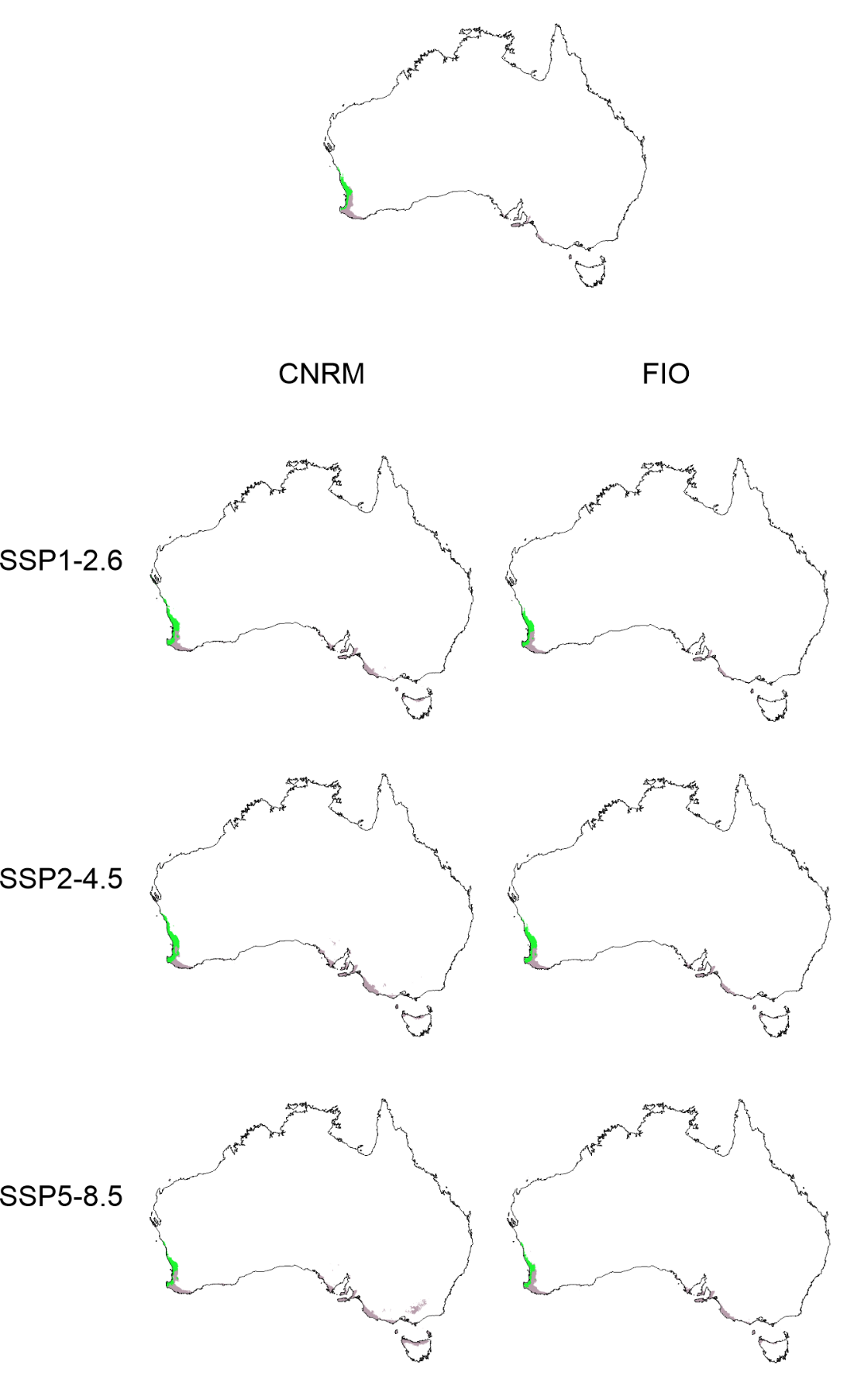


Figure 1. Overlap of potential range of Daviesia divaricata and Diuris magnifica. Areas of overlap marked with green, areas suitable only for orchid occurrence marked in grey. Upper map present current overlap between the species.

**Annex S13**. Overlap of potential range of pollinators and *Diuris brumalis.*


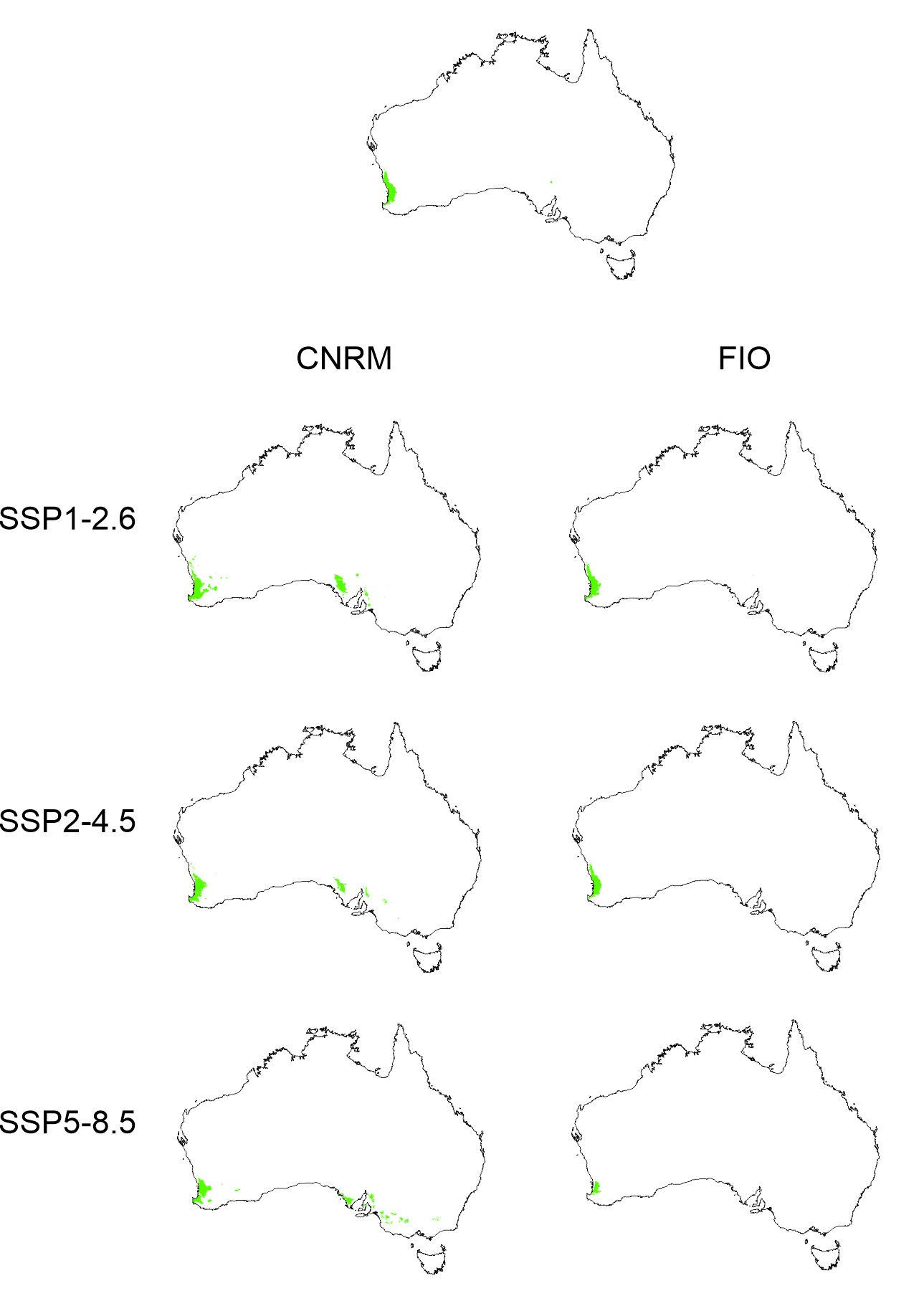


Figure 1. Overlap of potential range of Trichocolletes capillosus and Diuris brumalis. Areas of overlap marked with green, areas suitable only for orchid occurrence marked in grey. Upper map present current overlap between the species.


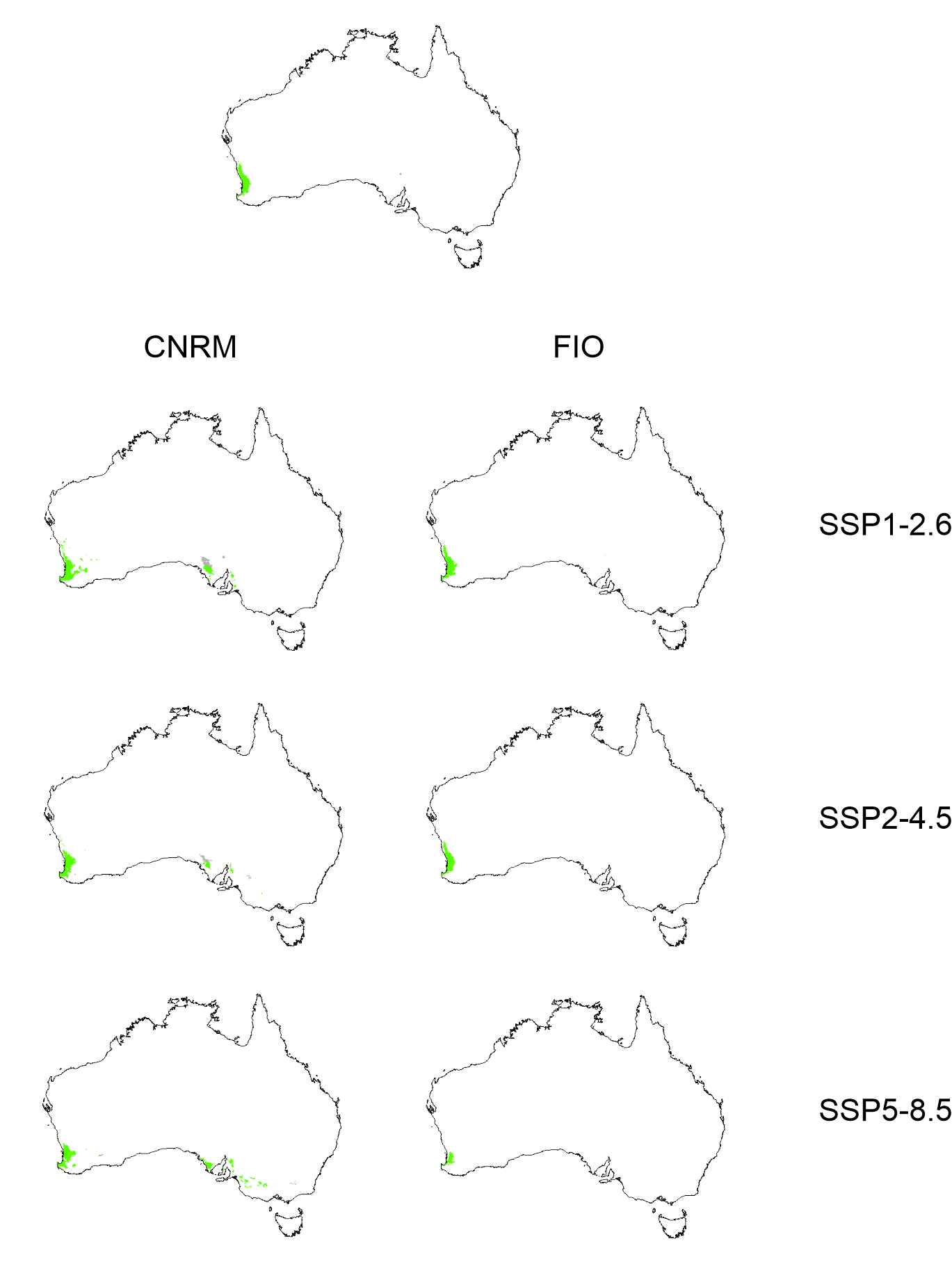


Figure 2. Overlap of potential range of Trichocolletes leucogenys and Diuris brumalis. Areas of overlap marked with green, areas suitable only for orchid occurrence marked in grey. Upper map present current overlap between the species.

**Annex S14**. Overlap of potential range of pollinators and *Diuris magnifica.*


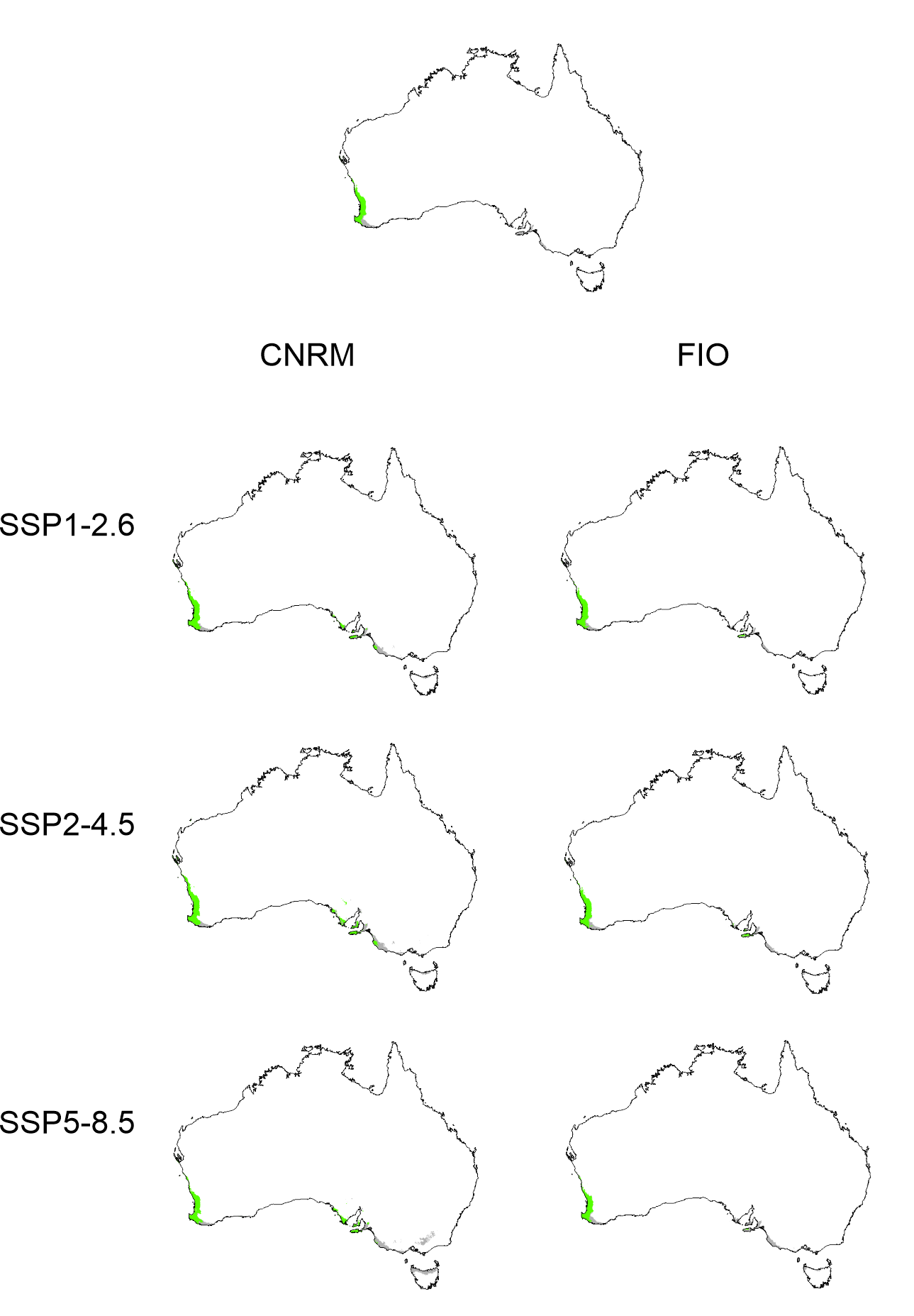


Figure 1. Overlap of potential range of Trichocolletes gelasinus and Diuris magnifica. Areas of overlap marked with green, areas suitable only for orchid occurrence marked in grey. Upper map present current overlap between the species.


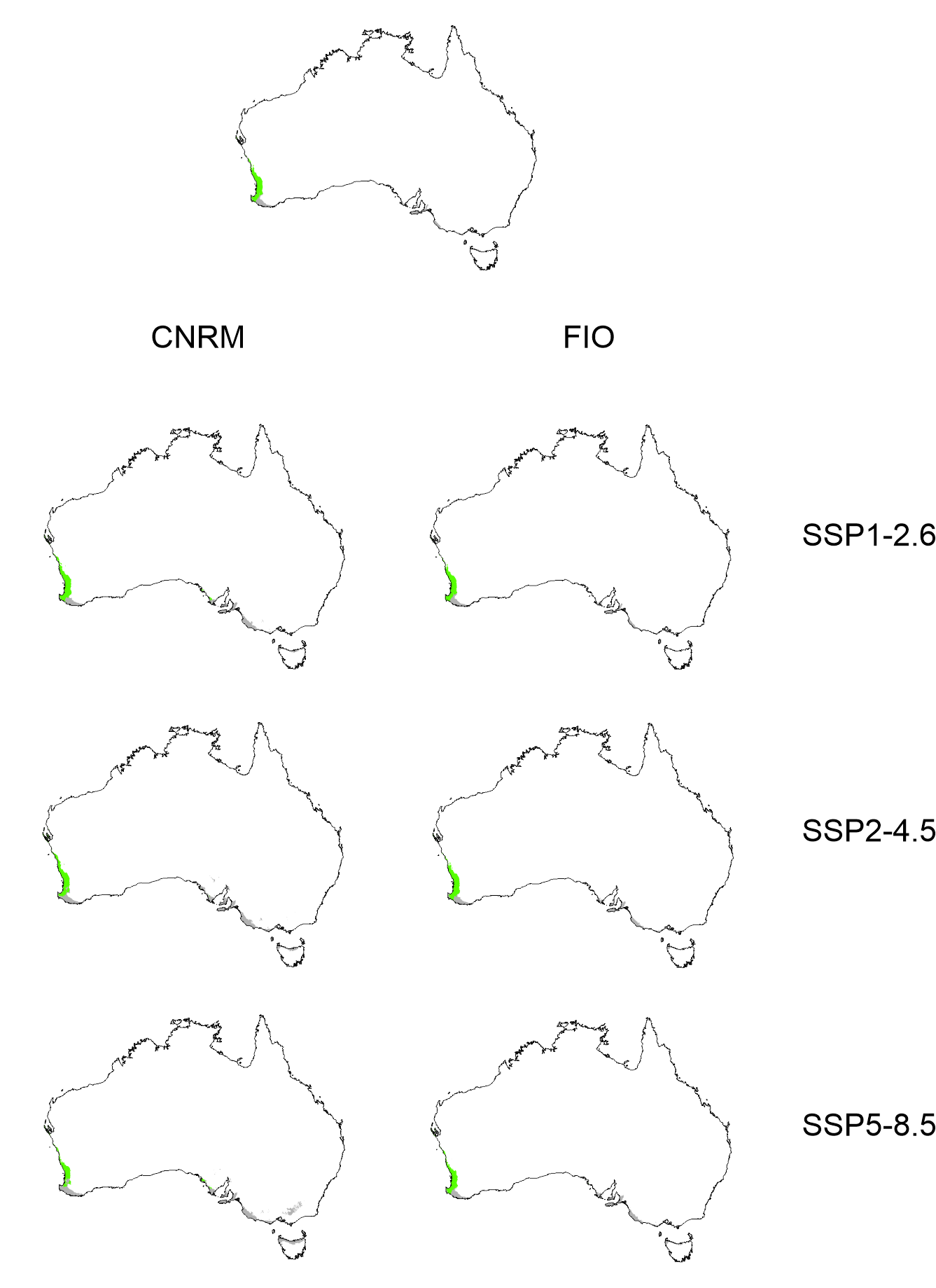


Figure 2. Overlap of potential range of Trichocolletes platyprosopis and Diuris magnifica. Areas of overlap marked with green, areas suitable only for orchid occurrence marked in grey. Upper map present current overlap between the species.
